# The distribution of parent‐reported autistic and subclinical ADHD traits in children with and without an autism diagnosis

**DOI:** 10.1002/jcv2.12259

**Published:** 2024-06-28

**Authors:** Tracey Chau, Jeggan Tiego, Louise E. Brown, Olivia J. Mellahn, Beth P. Johnson, Mark A. Bellgrove

**Affiliations:** ^1^ Turner Institute for Brain and Mental Health School of Psychological Sciences Monash University Clayton Victoria Australia; ^2^ School of Nursing, Midwifery & Paramedicine Curtin University Bentley Western Australia Australia

**Keywords:** attention‐deficit/hyperactivity disorder, autism, factor mixture modelling, latent structure, subclinical

## Abstract

**Background:**

Attention‐deficit/hyperactivity disorder (ADHD) traits often co‐occur in autistic children. The presence of subclinical ADHD traits can significantly impact upon different aspects of daily living. As such, understanding the distribution of these traits in autistic children may have important implications for the validity of diagnostic tools and subsequent intervention choices. This study builds on previous latent models of parent‐reported autistic and ADHD traits to propose a preliminary model of their distribution in two independent samples of autistic and neurotypical children.

**Methods:**

Factor mixture modelling was applied to caregiver responses to the Social Responsiveness Scale ‐ 2^nd^ edition and the Strengths and Weaknesses of ADHD Symptoms and Normal Behaviour Scale (SWAN) of participants aged 4–18 years who participated in one of two studies in Australia or in the United States.

**Results:**

A 2‐factor, 3‐class factor mixture model demonstrated the best fit to the data across both independent samples. The factors represented the latent constructs of ‘autism’ and ‘ADHD’. The latent classes represented subtypes of children with different levels of autistic traits, with higher levels of ADHD traits as autistic trait endorsement increased. Some sample‐specific differences were observed for each model's item thresholds and factor covariance matrices.

**Conclusions:**

Our findings suggest that the endorsement of subclinical ADHD traits tends to increase alongside autistic trait endorsement across neurotypical and autistic presentations. There may be clinical utility in routinely screening for ADHD traits in children with clinically elevated levels of autistic traits.

## Graphical abstract

Attention‐deficit/hyperactivity disorder (ADHD) traits often co‐occur in autistic children and the presence of subclinical ADHD traits can significantly impact upon different aspects of daily living. Our findings suggest that the endorsement of subclinical ADHD traits tends to increase alongside autistic trait endorsement across neurotypical and autistic presentations. There may be clinical utility in routinely screening for ADHD traits in children with clinically elevated levels of autistic traits.

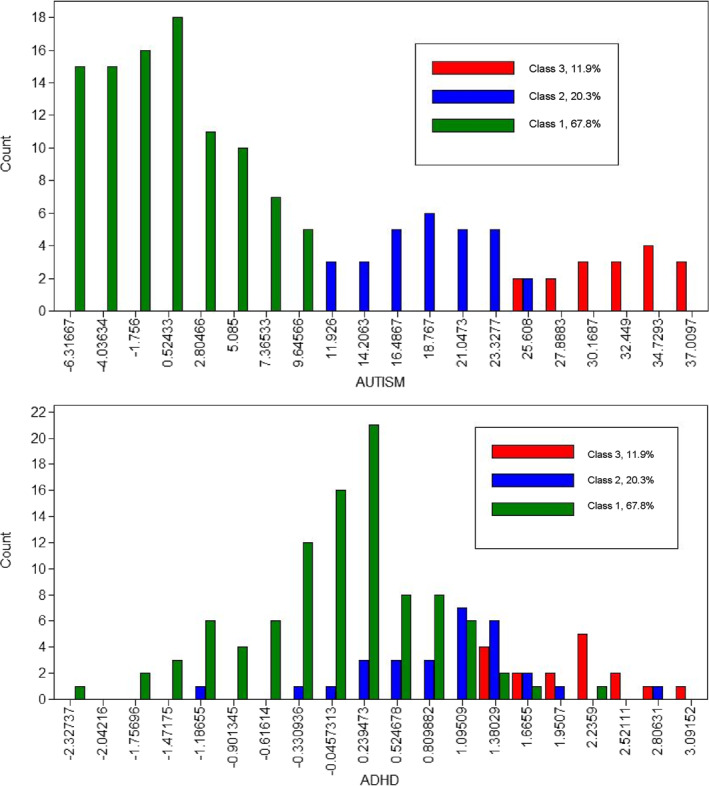

**Key points**

**What's known**

- Subclinical attention‐deficit/hyperactivity disorder (ADHD) traits are common in autistic children and can impact different domains of adaptive functioning. An understanding of their baseline distribution in autistic populations has yet to be established.

**What's new**

- Using Factor Mixture Modelling (FMM), we found that ‘autism’ and ‘ADHD’ were continuously distributed traits in two independent samples consisting of children with and without an autism diagnosis.
- The classes captured distinct but overlapping sections of the ‘autism’ continuum, suggesting that a dimensional conceptualisation of autistic traits is more consistent with the data than a categorical conceptualisation.
- Higher parent‐reported autistic traits corresponded to higher levels of parent‐reported subclinical ADHD traits.

**What's relevant**

- Routine screening for subclinical ADHD traits in children with high levels of autistic traits may be clinically meaningful.

## INTRODUCTION

Autism spectrum disorder (henceforth, ‘autism’) traits are understood to be continuously distributed in the population (Constantino, 2003) and share some neurobiological and clinical overlap with attention‐deficit/hyperactivity disorder traits (ADHD; Hoogman et al., 2022; van der Meer et al., 2017). However, it can take up to 3 years to identify co‐occurring, clinically significant ADHD traits after the initial autism diagnosis in children (Knott et alUnder review). The presence of ADHD traits can further impact how autistic children manage social (Harkins & Mazurek, 2023; Liu et al., 2021), cognitive (Schachar et al., 2023), and adaptive demands (Carpenter et al., 2022; Yerys et al., 2019) even if the combined number and impact of the traits do not meet the thresholds required for a formal ADHD diagnosis (i.e., these traits fall within a ‘subclinical’ range). However, their distribution in autistic children has yet to be established. Knowing this may clarify the relevant symptoms and thresholds that maximise the discriminant and predictive validity of ADHD diagnostic instruments (Antshel & Russo, 2019) and contribute towards bolstering the accessibility and relevance of current assessments and interventions for neurodivergent children.

Research suggests that co‐occurring ADHD traits are common in autistic individuals (Lee & Ousley, 2006; Leyfer et al., 2006; Simonoff et al., 2008; Sinzig et al., 2009). A recent paper by Hong et al. (2021) found that in a metropolitan clinic sample, just over 50% of autistic children aged 1.5–5 years of age exhibited moderate‐to‐high levels of ADHD traits. Given that autistic and ADHD traits are highly heterogeneous (Jeste & Geschwind, 2014; Karalunas & Nigg, 2020), quantitative modelling approaches that investigate potentially unobserved and more homogeneous subgroups of autistic and ADHD presentations (i.e., latent structure) can promote a shared understanding of similar experiences within the community and their relevant coping strategies. However, only one study has investigated the latent structure of ADHD traits in autistic children (Ghanizadeh, 2012; *N* = 68, mean age = 7.3 years). Exploratory factor analysis applied to the 12 autism and 18 ADHD‐related items of the Farsi version of the Children Symptom Inventory‐4 (Mohamadesmaiel & Alipour, 2002) revealed that the items loaded onto discrete autism and ADHD factors, respectively. Point estimates based on clinician‐administered diagnostic interviews also indicated that 53.8% of autistic children in their sample met the DSM‐IV diagnostic criteria for ADHD. Although these results provide meaningful support for the distinction between autistic and ADHD‐related traits in this population, there is an opportunity to expand upon these findings by modelling these traits in tandem and potentially identifying latent classes of presentations that may in the future inform further intervention (see Krakowski et al., 2021 for review).

Factor mixture modelling (FMM) is one such statistical technique that can facilitate this understanding. FMM is a hybrid of factor analytic and latent class techniques that can identify unique classes of participants who differ along different factors, within the one analysis (Clark et al., 2013). Previous work from our team (Chau et al., 2023) utilised FMM to establish and replicate a novel preliminary model of subclinical autistic traits in children with and without an ADHD diagnosis and demonstrated the superiority of FMM model fit to the data over individual factor analytic and latent class analyses in both discovery and replication samples. As such, these findings provide a strong, initial foundation for the use of FMM to investigate the presentation of subclinical ADHD traits in autistic and non‐autistic children.

Here, we aim to establish a preliminary model of parent‐reported autistic and subclinical ADHD traits in children with and without confirmed autism diagnoses. We sought to validate our findings by replicating the model in an independent dataset (Bauer & Curran, 2004). First, and consistent with previous findings (e.g., Ghanizadeh, 2012), we hypothesised that parent‐reported subscale measures of autistic and ADHD traits would load onto distinct (1) autism and (2) ADHD factors. Second, we predicted that FMM would produce two discrete classes of participants corresponding to (1) predominantly neurotypical children with low caregiver‐reported autistic traits and (2) predominantly autistic children with clinically elevated parent‐reported autistic traits (Deserno et al., 2022). Finally, we hypothesised that caregiver‐reported ADHD traits would be lower in the first class compared to the second class (e.g., Hong et al., 2021; van der Meer et al., 2012), but that there would be overlap in the distribution between the classes (Deserno et al., 2022).

## METHODS

### Research team

Both allistic and neurodivergent researchers with backgrounds in neurodevelopment (T.C., J.T., L.B., O.M., B.P.J., M.A.B.) and cross‐cultural psychology (T.C., L.B., J.T., M.A.B.) comprised the authorship team. We aimed to situate our study within a neurodiversity‐affirming framework by adopting a participatory action approach of actively engaging neurodivergent researchers during the data interpretation and manuscript preparation stages, and in leading discussions regarding the implications of the findings for the neurodivergent community (Bergold & Thomas, 2012).

### Participants

#### Recruitment

The discovery sample consisted of participants from the Monash Autism‐ADHD Genetics and Neurodevelopment (MAGNET) Project based in Melbourne, Australia (refer to Knott et al., 2021 for recruitment and assessment procedures).

Participants from the Healthy Brain Network (HBN) project formed the replication sample. This project is based in New York, United States of America (USA) and data are accessible through an online database (https://data.healthybrainnetwork.org/). Further details regarding the project's recruitment and testing processes are available in the Data Descriptor (Alexander et al., 2017).

#### Inclusion and exclusion criteria

From the MAGNET sample, only children enrolled as either neurotypical or autistic were included in the analysis. The HBN sample comprised of enrolled autistic children, those identified as autistic throughout their study participation, and neurotypical children. Across both samples, autistic children had their diagnoses verified against DSM‐5 criteria by expert clinicians from each respective project (refer to Knott et al., 2021; Alexander et al., 2017 for more information).

Exclusion criteria for each sample were (a) a diagnosis, or meeting standardised assessment cut‐offs for, a specific learning disorder, intellectual disability, and specific language impairment, (b) diagnoses of depressive or psychotic disorders and/or (c) diagnoses of another co‐occurring neurodevelopmental condition, including ADHD.

### Materials

#### Demographic variables

Participating children's age, sex (male or female), diagnostic status (autistic or neurotypical), and study site (HBN only) were collected during study enrolment. Diagnostic status was re‐confirmed at the conclusion of study participation.

#### Wechsler scales

Full‐scale intelligence (FSIQ) was obtained from the Wechsler Preschool & Primary Scale of Intelligence – Fourth Edition (WPPSI‐IV; Wechsler, 2012), Weschler Intelligence Scale for Children – Fourth or Fifth Edition (WISC‐IV or WISC‐V; Wechsler, 2014), or Weschler Abbreviated Scale of Intelligence – Second Edition (WASI‐II; Wechsler, 2011) depending on the child's age.

#### Social Responsiveness Scale – 2^nd^ edition (SRS‐2)

The school‐age SRS‐2 (Constantino & Gruber, 2012) is a standardised, parent‐report questionnaire that screens for social and non‐social traits characteristic of autism in children aged 4–18 years. Raw scores are converted to *T*‐scores on 5 subscales (Social Awareness, Social Cognition, Social Communication, Social Motivation, and Restricted Interests and Repetitive Behaviour) and 2 DSM‐5 compatible summary scales (DSM‐5 Social Communication and Interaction and DSM‐5 Restricted Interests and Repetitive Behaviour). Only the first five subscales were used in our analyses to allow for a detailed characterisation of the autism phenotype. The SRS‐2 utilises the following descriptors for specific *T*‐score ranges: ‘Normal’ (*T* ≤ 59), ‘Mild’ (60 ≤ *T* ≤ 65), ‘Moderate’ (66 ≤ *T* ≤ 75), and ‘Severe’ (*T* ≥ 76). In this paper, we will refer to the ranges as ‘Non‐autistic’, ‘Low’, ‘Moderate’, and ‘High’ respectively to be more consistent with the autistic community's language preferences (Bottema‐Beutel et al., 2021).

#### Strengths and Weaknesses of ADHD Symptoms and Normal Behaviour Scale (SWAN)

The SWAN (Swanson et al., 2001) is a parent‐report questionnaire that evaluates inattentive and hyperactive/impulsive behaviours in children that are consistent with ADHD. The two summary scales (Inattention and Hyperactivity/Impulsivity) represent the scaled sum of the items that constitute each domain. The summary scales each consist of 9 items that represent reworded versions of the DSM‐IV diagnostic criteria for ADHD. Example items include: ‘Gives close attention to detail and avoid careless mistakes’ (rewording of Criterion A1a, ‘often fails to give close attention to details or makes careless mistakes in schoolwork, work, or other activities’) and ‘Modulate verbal activity (control excess talking)’ (rewording of Criterion A2f, ‘Often talks excessively’). Each item is rated on a 7‐point Likert scale between −3 and +3, where a score of ‘0’ represents an ‘Average’ level of a particular strength or weakness. Scaled scores are also between −3 (‘far above average’) and +3 (‘far below average’), where higher scores (i.e., closer to +3) are more suggestive of clinically significant ADHD traits.

### Procedure

Parents completed the SRS‐2 and SWAN while participating children were administered the Wechsler scales as part of larger clinical and cognitive assessment batteries in each respective study. Where applicable, children participating in the MAGNET project were not required to withdraw from ADHD‐related medication (e.g., stimulants) prior to completing their cognitive assessment, while HBN participants were asked to withdraw from stimulant medication whenever possible. Across both studies, caregivers were requested to rate their child's behaviour while off‐medication.

### Statistical analyses

As the sizes of both samples were likely to exceed the minimum number required to identify the best fitting factor mixture models with small and large class separation (*N* = 75 and *N* = 25 respectively; Lubke & Neale, 2006), a formal power analysis was not conducted. Subsequent analyses proceeded as per previous work (Chau et al., 2023).

Data were analysed using Mplus (Version 8.7) and IBM's SPSS Statistics (Version 27). Boxplot graphs were generated using R. Where missing data could not be assumed to be missing at random (MAR) or missing completely at random (MCAR), data were imputed using multiple imputation with fully conditional specification (Enders, 2010; Van Buuren, 2007).

All discovery sample models were estimated using type ‘complex’ in Mplus to account for clustering by family (Muthén & Muthén, 2017). The nested nature of the HBN data by study site was ignored, because there were only five study sites and multilevel mixture models are recommended over single‐level models only when cluster number >50 (with cluster size >20) (Lee et al., 2018). Further, we attempted to calculate intraclass correlation coefficients (ICC) for each subscale by generating preliminary random effects linear mixed models with site as a level 2 variable. Results indicated that the covariance parameter for the random intercept was redundant for 6 of 7 subscales. For the remaining subscale (SRS‐2 Social Awareness Scale), the ICC = 0.03 which indicates that only 3% of the total variance of the subscale is attributable to between‐site variability. Taken together, both empirical and theoretical evidence supported proceeding with a single‐level analysis of the HBN data.

Subsequent analyses adhered to the FMM guidelines outlined by Clark et al. (2013). To establish comparison models and a priori upper bounds for the number of factors and classes of our FMM models, we generated a one‐factor common factor model and ran confirmatory factor analysis (CFA) and latent profile analyses (LPA) on the SRS‐2 and SWAN subscales. The CFA models were derived based on existing evidence that supports the questionnaires' ability to tap into the latent constructs of autism and ADHD respectively (Arnett et al., 2013; Frazier et al., 2014). Discriminant validity of the ADHD and autistic trait factors was also analysed (see Supporting Information S1: Appendix S1). Suggested interpretations of conventional fit statistics were used to evaluate CFA and LPA model fit (Bagozzi & Yi, 2012; Kline, 2015). Residual correlations were freely estimated to improve CFA model fit where empirically indicated based on modification indices, where consistent with theory, and if statistically significant when corrected for multiple comparisons using the Benjamini‐Hochberg procedure (Benjamini & Hochberg, 1995).

FMM analyses followed a methodical approach where the numbers of factors and classes in each model were increased by one class and/or factor at a time until their respective upper bounds were reached. The 4 main FMM variations (denoted FMM‐1 through FMM‐4; Clark et al., 2013) were estimated where possible (refer to Supporting Information S1: Appendix S2 for more information). We followed the guidelines by Nylund et al. (2007), as endorsed by Clark et al., 2013, to select the best fitting model among all CFA, LPA, and FMM models. In brief, this involves using the Bayesian Information Criterion (BIC) and Vuong‐Lo‐Mendell‐Rubin adjusted likelihood ratio test *p*‐value (VLMR*p*; Lo et al., 2001) to identify a subset of well‐fitting models, and evaluating the parametric bootstrapped likelihood ratio test *p*‐value (BLRT*p*; Mclachlan, 1987) in instances where the BIC and VLMR*p* disagree. Bayes factors (BF; Jeffreys, 1961) was also calculated to provide further empirical support for competing models. Importantly, the viability of competing models is also dependent on their substantive interpretation. To investigate whether there were any differences in age and FSIQ between the classes of the winning model, the model was re‐run with these two variables included as auxiliary variables using the BCH procedure (Asparouhov & Muthén, 2020).

Each participant's probabilistic class assignment was saved for subsequent class‐level analyses (more details available in Supporting Information S1: Appendix S3). A multivariate analysis of variance (MANOVA) was run where means of FMM‐derived latent variables could not be compared (i.e., FMM‐3 or FMM‐4), to investigate whether there were any class‐based differences in SRS‐2 and SWAN subscale ratings. Where homogeneity of error variances (i.e., Levene's Test) could not be assumed, Brown‐Forsythe tests (Brown & Forsythe, 1974) were performed with Benjamini‐Hochberg correction for a false discovery rate of 0.001. A smaller probability threshold was adopted because mixture models can result in smaller standard errors, biasing statistical comparisons between classes towards significance (Clark & Muthén, 2009). Post‐hoc Games‐Howell multiple comparisons tests were run to further investigate any significant group differences (Games et al., 1979).

#### Sensitivity analyses

The analyses were then repeated on a subset of each sample in which participants were drawn from an equivalent age interval (i.e., 5–17 years) to determine whether participants' age range impacted on the models' results.

## RESULTS

### Discovery sample – MAGNET data

A total of *N* = 164 (*n*_Neurotypical_ = 121, *n*_Autistic_ = 43) children enrolled in the MAGNET project, from 106 unique families, were included in this study. Participants were aged between 4 and 17 years (*M* = 8.64 years, *SD* = 2.95 years) and there were no significant differences in age between diagnostic groups (*t*(162) = −0.329, *p* = 0.743). There were slightly more females than males in the sample (53.7% female, *n* = 88). Females were more likely to be in the neurotypical group (χ^2^(1, *N* = 164) = 12.862, *p* < 0.001) with a small‐to‐medium effect size (Cramer's *V* = 0.280). Missing data was estimated to be 12.5%. Little's MCAR test was not significant (χ^2^(34, *N* = 164) = 31.347, *p* = 0.598), thus missing data were treated as ignorable (Little, 1988). All scores fell within the expected upper and lower bounds of each continuous measure (see Supporting Information S1: Table S1‐3 for descriptive statistics). Most of the continuous variables deviated significantly from normality (see Supporting Information S1: Table S4). Maximum likelihood with robust standard errors (MLR) estimation was used for subsequent analyses to account for the non‐normality and missingness of the data (Muthén & Muthén, 2017).

### Modelling results

The model fit statistics from the nested and non‐nested one‐factor common factor model, CFA, LPA, and FMM analyses in our discovery sample are summarised in Table 1. BLRT*p* values could not be generated for this sample due to the clustered nature of the data.

**TABLE 1:** Discovery sample: Factor analysis, latent profile analysis, and Factor Mixture Modelling results.

Model	Log likelihood	Entropy	AIC	BIC	VLMR *p*
Common Factor Model					
1 factor	−2764.954	‐	5571.908	5634.128	‐
1 factor (nested)	−2768.734	‐	5577.467	5636.724	‐
Confirmatory Factor Analysis					
2 factor	−2743.628	‐	5529.257	5591.476	‐
Latent Profile Analysis					
Equal Variances Across Classes					
1 class	−3241.632	‐	6511.263	6552.743	‐
2 classes	−2903.492	0.974	5850.983	5916.166	<0.001
3 classes	−2806.028	0.947	5672.057	5760.942	0.034
4 classes	−2754.975	0.921	5585.949	5698.537	0.320
5 classes	−2727.650	0.912	5547.300	5683.591	0.390
6 classes	−2702.511	0.908	5513.022	5673.016	0.260
Freely Estimated Variances Across Classes					
1 class	−3241.632	‐	6511.263	6552.743	‐
2 classes	−2883.757	0.967	5825.513	5911.436	<0.001
3 classes	−2761.523	0.941	5611.047	5741.412	0.002
4 classes	−2701.381	0.948	5520.762	5695.570	0.236
5 classes^					
6 classes	−2617.845	0.925	5413.690	5677.383	0.670
Factor Mixture Modelling					
1 factor, 2 classes					
FMM‐1	−2903.492	0.974	5850.983	5916.166	<0.001
FMM‐2	−2743.172	0.877	5532.344	5600.489	0.005
FMM‐3	−2728.290	0.910	5514.580	5600.503	0.367
FMM‐4	−2717.333	0.910	5504.666	5608.366	0.377
1 factor, 3 classes					
FMM‐1	−2813.021	0.945	5674.041	5745.150	0.018
FMM‐2	−2783.629	0.844	5527.258	5601.329	0.322
FMM‐3	−2704.644	0.929	5483.328	5592.954	0.340
FMM‐4	2676.711	0.891	5451.423	5596.602	0.151
2 factors, 2 classes					
FMM‐1^	−2743.628	‐	5529.257	5591.476	‐
FMM‐2	−2711.989	0.718	5479.978	5562.937	0.162
FMM‐3^					
FMM‐4^					
2 factors, 3 classes					
**FMM‐1**	**2711.325**	**0.848**	**5478.650**	**5561.610**	**0.584**
FMM‐2^					
FMM‐3^					
FMM‐4^					

*Note*: **Bold typeface** = best fitting model; # = Loglikehood was not replicated; ^ = Model misspecified.

Abbreviations: AIC, Akaike Information Criterion; BIC, Bayesian Information Criterion; BLRT*p*, Bootstrapped Likelihood Ratio Test *p*‐value; FMM, Factor Mixture Model; *p*, probability value of the test statistic; VLMR*p*, Vuong‐Lo‐Mendell‐Rubin *p*‐value.

#### Confirmatory factor analysis

Two CFA models were obtained: (1) an original model and (2) a modified model (see Supporting Information S1: Figures S1–2). The inclusion of post‐hoc modifications in model (2) improved model fit across most goodness‐of‐fit indices (Bagozzi & Yi, 2012). All bivariate relationships were also appropriately reproduced following these modifications (see Tables S5‐6; all correlational residuals (ε) < |0.1|; Kline, 2015). Standardised and unstandardised factor loading coefficients for this model are shown in Supporting Information S1: Table S7. Discriminant validity between our two factors was indicated, as 1.00 was outside of the 95% confidence interval for the factor intercorrelation [0.603, 0.832], and the squared correlation (51.6%) was less than the average variance extracted (82.1% and 74.5% for the autistic and ADHD traits factors, respectively). This revealed that more variance was explained by each factor in its indicators than variance shared between them. Additionally, the modified two‐factor CFA model demonstrated a better fit to the data than the nested one‐factor model (Satorra‐Bentler scaled difference chi‐square statistic [TRd] = 36.869, *p* < 0.001, Cohen's *d* = 1.077).

#### Latent profile analyses

Based on the BIC and VLMR*p* values displayed in Table 1, the ideal class upper bound was 3 for all FMM models.

#### Factor mixture modelling

Based on the CFA and LPA results, models with up to 2 factors and 3 classes were fit. Following the model selection advice by Nylund et al. (2007), we began with the 2‐factor, 3‐class FMM‐1 model as it had the lowest BIC. However, the VLMR*p* value was significant indicating that the *k*‐1 class model (i.e., 2‐factor, 2‐class FMM‐1) should be favoured. Given that the *k*‐1 class model was misspecified, the original 2‐factor, 3‐class FMM‐1 model was retained as the best‐fitting model. Bayes factor strongly supported this model over the CFA model (BF > 100; Kass & Raftery, 1995).

Further class‐level analyses were conducted as the model demonstrated acceptable class separation (entropy >0.80; Greenbaum et al., 2005). All neurotypical children were allocated to either Class 1 (*n* = 92 of 104; 88.5%) or Class 2 (*n* = 12 of 104; 11.5%). Autistic children were mostly assigned to Class 2 and Class 3 (*n* = 17 of 39 in both classes; 43.6% each). There were no significant effects of sex across classes (χ^2^(2, *N* = 143) = 4.292, *p* = 0.117). Tables S8‐9 summarise the auxiliary variable analyses. There was no significant difference between classes in age (χ^2^(2, *N* = 164) = 1.736, *p* = 0.420). Participants in Class 3 had significantly higher mean FSIQ than those in Class 2 (χ^2^(2, *N* = 164) = 11.659, *p* = 0.001), but not Class 1 (χ^2^(2, *N* = 164) = 3.648, *p* = 0.056). There was no significant difference in mean FSIQ between Class 1 and Class 2 (*p* = 0.815). All mean FSIQs fell within less than one standard deviation of the Wechsler index score mean (*M* = 100, *SD* = 15).

Table 2 displays the mean SRS‐2 and SWAN subscale scores by class. Participants in Class 3 were characterised by high levels of autistic traits and elevated/above‐average levels of ADHD traits. Class 2 was defined by low‐to‐moderate levels of autistic traits and average levels of ADHD traits. Finally, children in Class 1 were rated as displaying non‐autistic levels of autistic traits and average‐to‐below average levels of ADHD traits.

**TABLE 2:** Discovery sample: Summary of SRS‐2 and SWAN subscale scores by class assignment.

	Class 1 (n = 97) M (SD)	Class 2 (n = 29) M (SD)	Class 3 (n = 17) M (SD)
*SRS‐2*			
Social awareness	50.42 (9.915)	68.00 (10.090)	82.13 (6.589)
Social cognition	47.33 (6.502)	68.00 (7.198)	79.47 (7.080)
Social communication	48.33 (6.629)	67.95 (4.477)	83.47 (5.755)
Social motivation	48.96 (7.389)	64.50 (10.591)	77.80 (6.971)
Restricted interests and repetitive behaviour	48.10 (5.784)	65.05 (7.214)	83.40 (6.663)
*SWAN*			
Inattention	−0.330 (0.929)	0.523 (0.912)	1.763 (0.566)
Hyperactivity‐impulsivity	−0.362 (1.040)	0.409 (0.585)	1.139 (1.404)

Abbreviations: *M*, mean; *n*, size of subsample; *SD*, standard deviation; SRS‐2, Social Responsiveness Scale (2^nd^ Edition); SWAN, Strengths and Weaknesses of ADHD and Normal Behaviour Scale.

**FIGURE 1:**
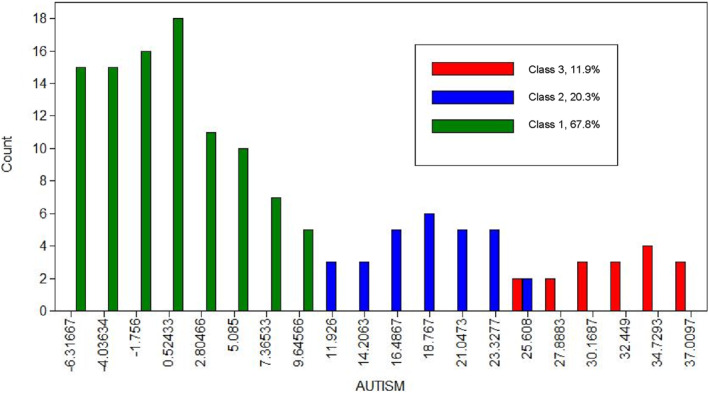
Discovery Sample: ‘Autism’ Factor Score Distribution by Factor Mixture Modelling Class Assignment *x*‐axis depicts factor scores along ‘Autism’ factor; *y*‐axis displays the number of participants in each factor score bin.

**FIGURE 2:**
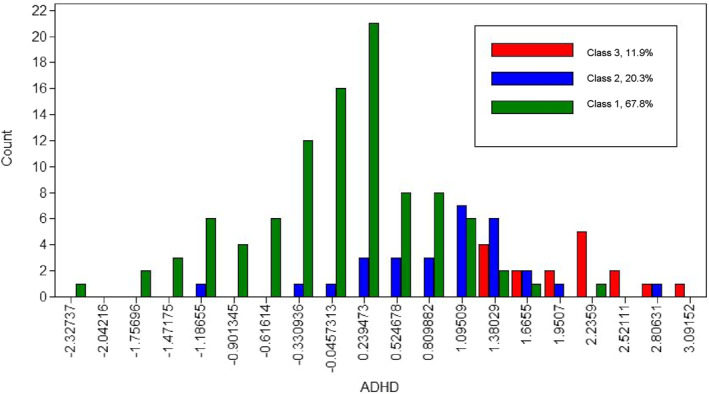
Discovery Sample: ‘ADHD’ Factor Score Distribution by Factor Mixture Modelling Class Assignment *x*‐axis depicts factor scores along ‘attention‐deficit/hyperactivity disorder (ADHD)’ factor, factor scores are standardised with a mean of zero and standard deviation of 1; *y*‐axis displays the number of participants in each factor score bin.

Figures 1 and 2 displays the distribution of factor scores along the ‘Autism’ and ‘ADHD’ factors separated by class. Children rated as displaying higher autistic traits were also reported as exhibiting higher ADHD traits. Further visual inspection of both graphs initially suggests a categorical separation of classes along the ‘Autism’ factor and a continuous (or dimensional) distinction along the ‘ADHD’ factor. However, the proportion of autistic and neurotypical children allocated to each class indicates that these seemingly categorical separations are, in fact, more dimensional in nature and the diagnostic groups are not cleanly represented by the classes.

### Independent sample – HBN project

A total of *N* = 418 (*n*_Neurotypical_ = 351, *n*_Autistic_ = 67) children enrolled in the HBN project met the inclusion criteria for our study. Participants were aged between 5 and 18 years (*M* = 9.96 years, *SD* = 3.17 years) and autistic participants were significantly younger than the neurotypical children (*t*(416) = −2.101, *p* = 0.036) with a small effect size (Cohen's *d* = 0.280). There were slightly more males than females in the sample (56.5% male, *n* = 236). Females were more likely to be in the neurotypical group (χ^2^(1, *N* = 418) = 12.545, *p* < 0.001), but the associated effect size was also small (Cramer's *V* = 0.173). Missing data was estimated to be 3.29%. Little's MCAR Test was significant (χ^2^(54, *N* = 418) = 108.973, *p* < 0.001), indicating that data could not be assumed to be MAR (Little, 1988). As such, the data were multiply imputed (50 iterations, 20 imputations) and the pooled dataset used for the remaining analyses.

Supporting Information S1: Tables S10‐12 display the descriptive statistics for the continuous variables pre‐imputation. Post‐imputation statistics can be found in Supporting Information S1: Tables S13‐15. All variables were non‐normally distributed (see Supporting Information S1: Table S16). Consistent with analyses of the discovery sample, MLR estimation was used for subsequent analyses.

### Modelling results

The model fit statistics from the nested and non‐nested one‐factor common factor model, CFA, LPA, and FMM analyses are summarised here (Table 3).

**TABLE 3:** Replication sample: Factor analysis, latent profile analysis, and Factor Mixture Modelling results.

Model	Log likelihood	Entropy	AIC	BIC	VLMR *p*	BLRT*p*
Common Factor Model						
1 factor	−8218.671	‐	16,479.343	16,564.088	‐	‐
1 factor (nested)	−8167.351	‐	16,374.792	16,455.412	‐	‐
Confirmatory Factor Analysis						
2 factor	−8132.718	‐	16,307.436	16,392.181	‐	‐
2 factor (modified)	−8097.946	‐	16,245.892	16,346.779	‐	‐
Latent Profile Analysis						
Equal Variances Across Classes						
1 class	−9176.787	‐	18,381.573	18,438.070	‐	‐
2 classes	−8578.314	0.948	17,200.627	17,289.408	<0.001	<0.001
3 classes	−8335.482	0.883	16,730.964	16,852.028	0.773	<0.001
4 classes	−8196.561	0.888	16,469.123	16,622.471	0.114	<0.001
5 classes	−8103.116	0.882	16,298.233	16,483.865	0.037	<0.001
6 classes	−8042.616	0.894	16,193.231	16,411.147	0.028	<0.001
Freely Estimated Variances Across Classes						
1 class	−9176.787	‐	18,381.573	18,438.070	‐	‐
2 classes	−8410.261	0.898	16,878.523	16,995.552	<0.001	<0.001
3 classes	−8105.753	0.904	16,299.506	16,477.067	<0.001	<0.001
4 classes	−7976.025	0.894	16,070.050	16,308.144	0.004	<0.001
5 classes	−7879.667	0.893	15,907.335	16,205.960	0.011	<0.001
6 classes	−7801.425	0.903	15,780.850	16,140.008	0.031	<0.001
Factor Mixture Modelling						
1 factor, 2 classes						
FMM‐1	−8578.314	0.948	17,200.628	17,289.408	<0.001	<0.001
FMM‐2	−8171.792	0.874	16,389.583	16,482.399	<0.001	<0.001
FMM‐3	−8064.114	0.887	16,186.228	16,303.257	<0.001	<0.001
FMM‐4	−8038.862	0.864	16,147.723	16,288.965	<0.001	<0.001
1 factor, 3 classes						
FMM‐1	−8353.281	0.893	16,754.561	16,851.413	0.153	0.667
FMM‐2	−8160.954	0.835	16,371.907	16,472.794	0.032	1.00
FMM‐3	−7969.686	0.918	16,013.372	16,162.685	<0.001	<0.001
FMM‐4	−7954.632	0.901	16,007.265	16,205.003	0.160	<0.001
1 factor, 4 classes						
FMM‐1	−8235.967	0.881	16,523.933	16,628.856	0.189	1.00
FMM‐2#						
FMM‐3	−7931.868	0.925	15,953.736	16,135.333	0.273	<0.001
FMM‐4#						
1 factor, 5 classes						
FMM‐1	−8184.687	0.867	16,425.373	16,538.367	<0.001	0.500
FMM‐2	−8155.001	0.771	16,368.002	16,485.031	0.416	1.00
FMM‐3	−7906.070	0.921	15,918.139	16,132.020	0.168	<0.001
FMM‐4^						
1 factor, 6 classes						
FMM‐1	−8164.961	0.876	16,389.923	16,510.987	0.187	0.375
FMM‐2^						
FMM‐3	−7886.872	0.915	15,896.744	16,141.909	0.536	<0.001
FMM‐4#						
2 factors, 2 classes						
FMM‐1^						
FMM‐2	−8040.808	0.687	16,137.615	16,250.609	0.240	<0.001
FMM‐3	−8008.546	0.754	16,083.092	16,216.263	<0.001	<0.001
FMM‐4^						
2 factors, 3 classes						
FMM‐1^						
FMM‐2^						
**FMM‐3**	**−7935.105**	**0.924**	**15,958.210**	**16,136.771**	**<0.001**	**<0.001**
FMM‐4^						
2 factors, 4 classes						
FMM‐1^						
FMM‐2^						
FMM‐3^						
FMM‐4^						
2 factors, 5 classes						
FMM‐1^						
FMM‐2^						
FMM‐3^						
FMM‐4^						
2 factors, 6 classes						
FMM‐1^						
FMM‐2^						
FMM‐3^						
FMM‐4^						

*Note*: **Bolded typeface** = best fitting model; # = Loglikehood was not replicated; ^ = Model misspecified.

Abbreviations: AIC, Akaike Information Criterion; BIC, Bayesian Information Criterion; BLRT*p*, Bootstrapped Likelihood Ratio Test *p*‐value; FMM, Factor Mixture Model; *p*, probability value of the test statistic; VLMR*p*, Vuong‐Lo‐Mendell‐Rubin *p*‐value.

#### Confirmatory factor analysis

We produced two CFA models: (1) an original model, and (2) a modified model (see Supporting Information S1: Figures S3–4). The inclusion of post‐hoc modifications in model (2) improved model fit across most goodness‐of‐fit indices (Bagozzi & Yi, 2012) and ensured that all bivariate relationships were appropriately reproduced (see Supporting Information S1: Tables S17‐18; all correlation residuals (ε) < |0.1|; Kline, 2015). Supporting Information S1: Table S19 displays the regression coefficients for both models. Once again, 1.00 was outside of the 95% confidence interval for the factor intercorrelation [0.492, 0.646], and the squared correlation (32.4%) was less than the average variance extracted (77.9% and 70.1% for the autistic and ADHD traits factors, respectively). These results indicated adequate discriminant validity between the ‘Autism’ and ‘ADHD’ factors. Furthermore, comparison between the nested one‐factor model and the modified two‐factor CFA model indicated that the two‐factor model demonstrated a better fit to the data (TRd = 133.400, *p* < 0.001, Cohen's *d* = 1.369).

#### Latent profile analyses

The ideal upper bound for the number of classes was 6 for all FMM models as indicated by the BIC, VLMR*p*, and BLRT*p* values in Table 3.

#### Factor mixture modelling

Based on these results, models with up to 2 factors and 6 classes were fit. Once again, we started our model selection process with the model with the lowest BIC value: the 1‐factor, 5‐class FMM‐3 model. The VLMR*p* value (>0.05) favoured the *k*‐1 class model, which also had the next lowest BIC value. Repeating the process with the 1‐factor, 4‐class FMM‐3 model, we observed that the VLMR*p* value (>0.05) once again favoured the *k*‐1 class model (i.e., 1‐factor, 3‐class FMM‐3). However, this model did not have the lowest BIC value of all remaining models; this belonged to the 2‐factor, 3‐class FMM‐3 model. Bayes' factor comparison strongly favoured this competing model (BF > 100; Kass & Raftery, 1995) model over the 1‐factor, 3‐class FMM‐3 model. On a balance of all fit statistics and substantive interpretability, the 2‐factor, 3‐class FMM‐3 model was selected as the best fitting model. This represents a partial replication of the discovery sample's results, where a 2‐factor, 3‐class FMM‐1 model was determined as the best‐fitting model.

Again, further class‐level analyses were conducted as the model demonstrated acceptable class separation (entropy >0.80; Greenbaum et al., 2005). Most neurotypical children were allocated to Class 1 (*n* = 75 of 351, 21.4%) and Class 2 (*n* = 263 of 351, 74.9%) and most autistic children were assigned to Class 2 (*n* = 18 of 67, 26.9%) and Class 3 (*n* = 39 of 67, 58.2%). A small‐to‐medium effect of sex was detected between classes (χ^2^(2, *N* = 418) = 24.031, *p* < 0.001; Cramer's *V* = 0.240). More females than males were allocated to Class 1, whereas more males than females were allocated to Class 2 and 3. It is likely that this reflects the sample's sex characteristics (i.e., females more likely to be enrolled as neurotypical). Supporting Information S1: Tables S20‐21 summarise the auxiliary variable analyses. There was no significant difference between classes in age (χ^2^(2, *N* = 351) = 1.692, *p* = 0.429). Participants in Class 1 and Class 2 had equivalent mean FSIQ scores that were both significantly higher than those in Class 3 (Class 1 vs. Class 3: χ^2^(2, *N* = 351) = 8.462, *p* = 0.004; Class 2 versus Class 3: χ^2^(2, *N* = 351) = 5.316, *p* = 0.021). All mean FSIQs fell within less than one standard deviation of the Wechsler index score mean (*M* = 100, *SD* = 15).

Table 4 displays the mean SRS‐2 and SWAN subscale scores by class. Participants in Class 1 and Class 2 were characterised by non‐autistic levels of autistic traits and below average and average levels of ADHD traits respectively. Children in Class 3 were rated as displaying low‐to‐moderate levels of autistic traits and average‐to‐above average levels of ADHD traits.

**TABLE 4:** Replication sample: Summary of SRS‐2 and SWAN subscale scores by class assignment.

	Class 1 (*n* = 85) *M (SD)*	Class 2 (*n* = 281) *M (SD)*	Class 3 (*n* = 52) *M (SD)*
*SRS‐2*			
Social awareness	47.726 (10.325)	52.957 (8.252)	67.096 (10.485)
Social cognition	48.396 (10.109)	49.810 (7.388)	69.779 (10.525)
Social communication	49.576 (10.228)	49.523 (6.975)	69.641 (10.422)
Social motivation	50.475 (10.799)	49.731 (8.021)	61.266 (12.020)
Restricted interests and repetitive behaviour	47.481 (6.865)	48.130 (5.881)	75.343 (7.981)
SWAN			
Inattention	−1.278 (1.097)	−0.039 (0.886)	0.878 (0.955)
Hyperactivity‐impulsivity	−2.110 (0.648)	0.065 (0.569)	0.630 (0.946)

Abbreviations: *M*, mean; *n*, size of subsample; *SD*, standard deviation; SRS‐2, Social Responsiveness Scale (2^nd^ edition); SWAN, Strengths and Weaknesses of ADHD and Normal Behaviour Scale.

Supporting Information S1: Table S22 displays the MANOVA results comparing the mean subscale scores of participants across classes. The results are described here in brief. As Box's *M* was significant (*p* < 0.001), Pillai's Trace is reported as it is robust to violations of the multivariate homogeneity of variance assumption (Tabachnick & Fidell, 2013). Results from Levene's test indicated that the univariate homogeneity of error variance assumption was also violated for all subscales. As such, univariate Brown‐Forsythe tests were undertaken with Benjamini‐Hochberg corrections. Results indicated a large multivariate (Pillai's trace = 1.392, *F*(14, 820) = 133.942, *p* < 0.001, partial *η* ^2^ = 0.696) and univariate effects of class assignment on all subscales (see Supporting Information S1: Table S23 for more details). The largest effect of class was detected on the Hyperactivity‐Impulsivity scale (partial *η* ^2^ = 0.678; see Supporting Information S1: Table S15 for more details). Post‐hoc Games‐Howell analyses showed that while those in Class 3 demonstrated significantly higher ratings than participants in both Class 1 and Class 2 across all subscales (all *p* < 0.001; range for Hedges' |*g|*: [0.952, 3.520]), Class 2 demonstrated significantly higher ratings than Class 1 on the SRS‐2 Social Awareness subscale, and both SWAN summary scales only (all *p* < 0.001; range for Hedges' |*g*|: [0.640, 3.690]).

**FIGURE 3:**
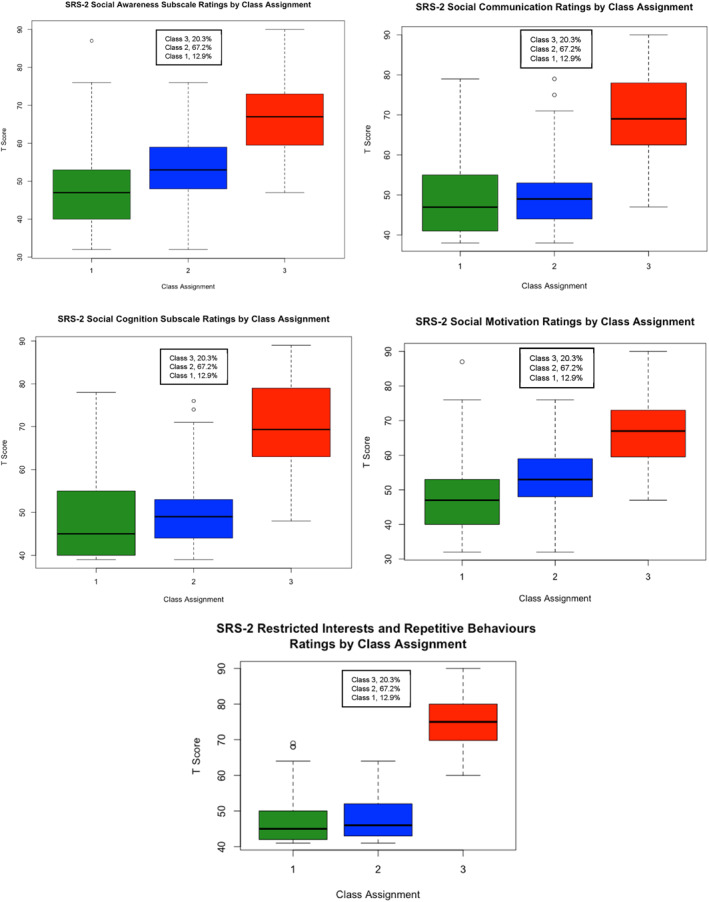
Replication sample: Boxplots of social responsiveness scale (2^nd^ edition; SRS‐2) subscale ratings by Factor Mixture Modelling class assignment.

**FIGURE 4:**
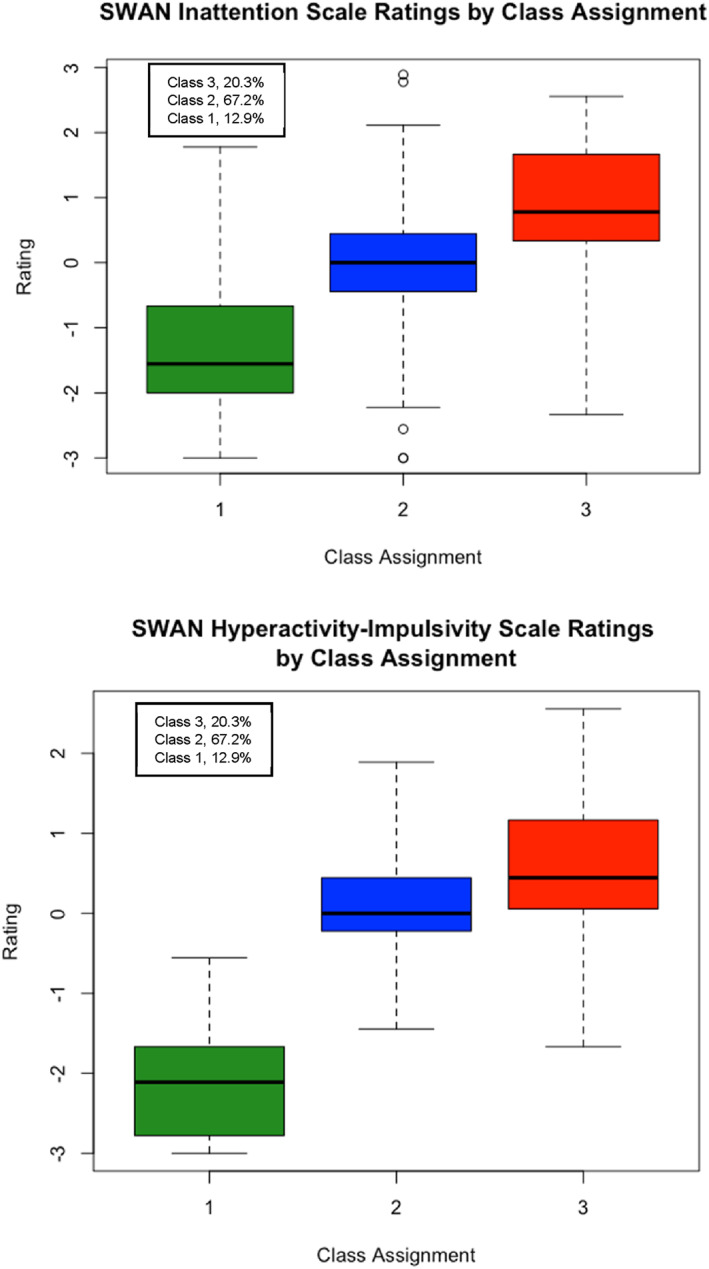
Replication sample: Boxplots of SWAN summary scale ratings by Factor Mixture Modelling class assignment.

Figures 3 and 4 display the boxplots of the distribution of SRS‐2 and SWAN subscale score frequencies by class assignment. Visual inspection of the boxplots once again suggested that children rated as displaying higher levels of autistic traits also displayed higher levels of ADHD traits, but that the distinction between the classes along the SRS‐2 scales were dimensional rather than categorical in nature. Similarly, greater overlap between the classes were observed on the SWAN subscales which points to a dimensional distribution of subclinical ADHD traits.

### Sensitivity analyses

Interpretation of the results from the sensitivity analyses did not differ from those of the original analyses (see full results in Supporting Information S1: Appendix S4). As such, the analyses undertaken on data from the original samples were retained so that the representation of autistic and neurotypical children across childhood and adolescence was maximised.

## DISCUSSION

ADHD traits commonly co‐occur in autistic children, and their presence can confer the need for additional scaffolding to support the individual child's success (Carpenter et al., 2022; Yerys et al., 2019). Our identification here of latent, homogeneous subgroups of autistic children, with differing levels of subclinical ADHD traits, may help to inform these intervention decisions. Following from previous work (Chau et al., 2023), our study presents novel factor mixture models of parent‐reported autistic and subclinical ADHD traits in children aged 4–18 years with and without an autism diagnosis. The findings indicate that subclinical ADHD traits tend to increase as autistic trait endorsement increases, and that there may be clinical utility in the routine screening of subclinical ADHD traits in children with clinically elevated levels of autistic traits.

As hypothesised, we demonstrated that autistic and ADHD traits coalesced around distinct factors as estimated by the SRS‐2 and SWAN, respectively. Autistic traits were also continuously distributed in both samples. Next, we partially replicated a 2‐factor, 3‐class factor mixture model in two independent samples based on responses to the SRS‐2 and SWAN. Although this differs slightly from our original hypothesis of a 2‐class model, the three classes of participants did differ along the latent factors of ‘autism’ and ‘ADHD’ in the expected direction and captured distinct, but overlapping, subgroups of children. Indeed, and in contrast to existing findings (e.g., Deserno et al., 2022; Krakowski et al., 2021), we found that the classes consisted mostly of both neurotypical and autistic children suggesting that dimensional conceptualisations of autistic have more validity than categorical conceptualisations in the current dataset. The distribution of scores along the ‘ADHD’ factor also remained continuous across all the classes and supports a dimensional conceptualisation of ADHD traits in neurodivergent and neurotypical children as suggested previously (Deserno et al., 2022).

Across both samples, mean subclinical ADHD trait endorsement increased in line with autistic trait endorsement. This indicates that in children with and without an autism diagnosis, children who exhibit higher levels of autistic traits also tend to exhibit higher levels of subclinical ADHD traits. As such, the routine screening of subclinical ADHD traits in children with clinically elevated levels of autistic traits may usefully contribute to subsequent clinical decision‐making, particularly given the demonstrated impact of subclinical ADHD traits on adaptive functioning in autistic children (Yerys et al., 2019). Additionally, when considered alongside previous work (Chau et al., 2023), the findings highlight a potential difference in the distribution of subclinical autistic and ADHD traits in autistic children compared to children diagnosed with ADHD. Further investigation into these differences may help to improve the validity and reliability of autism and ADHD screening instruments in neurodivergent populations.

### Limitations and future directions

It would be worthwhile for future studies to attempt to replicate our models in other independent samples. In an FMM‐3 model, the factor covariance matrix and item thresholds are freely estimated (i.e., allowed to vary between classes) whereas they are held invariant in an FMM‐1. This suggests that the best‐fitting factor mixture models identified greater homogeneity amongst neurotypical and amongst autistic participants in the discovery sample (i.e., FMM‐1 model) and more heterogeneity in the replication sample (i.e., FMM‐3 model). The heterogeneity may be attributable to parental differences in child ratings independent of the latent variable (Clark et al., 2013). Nevertheless, the difference between FMM iterations raises queries as to whether our samples have captured different aspects of the broader heterogeneity within autism, or whether this represents methodological artefact of our sample selection. Indeed, the significant effect of sex on class assignment in the replication sample, but not the discovery sample, points to possible variability between the rating thresholds for autistic and/or ADHD traits used by parents of male children as compared to female children. This pattern has been detected before (e.g., Duvekot et al., 2017; Little et al., 2017). Additionally, the exclusion criteria for our samples may have restricted our ability to capture other subsets of the autistic population, such as those who experience co‐occurring mood, intellectual, and/or learning challenges. Conducting the modelling analyses on a larger, more representative population sample of autistic and neurotypical children may help to address these limitations.

Future studies should also include child‐report measures to also capture the internal experience of autism (e.g., sensory integration differences, ‘flow’ states, masking tendencies; McQuaid et al., 2022; Dupuis et al., 2022; Schaaf et al., 2022) and ADHD (e.g., hyperfocus, rejection sensitivity; Ashinoff & Abu‐Akel, 2021; Bondü & Esser, 2015), and determine whether the classes differ depending on the informant. The stratification of analyses by age could help to account for potential developmental differences in the distribution of autistic and ADHD traits in this population. Finally, a longitudinal evaluation of these classes may yield clinically meaningful insights into their predictive validity for outcomes such as future diagnostic conversion, quality of life, and adaptive functioning. It is possible that there may be differences on these outcomes between autistic children who received autism‐only interventions and those who received intervention that also accounted for their subclinical ADHD traits.

## CONCLUSION

Across two independent samples, our study demonstrated that higher parent‐reported autistic traits corresponded to higher parent‐reported subclinical ADHD traits in children with and without an autism diagnosis. The findings support a dimensional rather than a categorical conceptualisation of autism in relation to neurotypical presentations. Our results suggest that there may be clinical importance in the routine screening of subclinical ADHD traits in children with high levels of autistic traits.

## AUTHOR CONTRIBUTIONS

**Tracey Chau**: Conceptualization; Formal analysis; Investigation; Project administration; Writing – original draft; Writing – review & editing. **Jeggan Tiego**: Conceptualization; Formal analysis; Methodology; Supervision; Writing – original draft; Writing – review & editing. **Louise E. Brown**: Methodology; Writing – review & editing. **Olivia J. Mellahn**: Methodology; Project administration; Writing – review & editing. **Beth P. Johnson**: Data curation; Project administration; Supervision; Writing – review & editing. **Mark A. Bellgrove**: Conceptualization; Data curation; Funding acquisition; Investigation; Methodology; Supervision; Writing – original draft; Writing – review & editing.

## CONFLICT OF INTEREST STATEMENT

The authors have declared that they have no competing or potential conflicts of interest.

## ETHICAL CONSIDERATIONS

The MAGNET Project was approved by Monash University Human Research Ethics Committee (HREC; CF16/1537‐2016000806), Department of Education and Training Victoria HREC (2017_003570), and Monash Health HREC (RES‐19‐0000‐372A). The HBN project was approved by the Chesapeake Institutional Review Board. Written consent was obtained from participating parents or legal guardians, and any participants aged 18 years or older, and verbal (MAGNET) or written (HBN) assent was obtained from children younger than 18 years.

## Supporting information

Supporting Information S1

## Data Availability

The data that support the findings of this study from the Monash Autism‐ADHD Genetics and Neurodevelopment Study are available from the corresponding author upon reasonable request. The data from the Healthy Brain Network that support the findings of this study are openly available in the Child Mind Institute Healthy Brain Network data portal.

## References

[jcv212259-bib-0001] Alexander, L. M. , Escalera, J. , Ai, L. , Andreotti, C. , Febre, K. , Mangone, A. , Vega‐Potler, N. , Langer, N. , Alexander, A. , Kovacs, M. , Litke, S. , O'hagan, B. , Andersen, J. , Bronstein, B. , Bui, A. , Bushey, M. , Butler, H. , Castagna, V. , Camacho, N. , … Milham, M. P. (2017). An open resource for transdiagnostic research in pediatric mental health and learning disorders. Scientific Data, 4(1), 170181. 10.1038/sdata.2017.181 29257126 PMC5735921

[jcv212259-bib-0002] Antshel, K. M. , & Russo, N. (2019). Autism spectrum disorders and ADHD: Overlapping phenomenology, diagnostic issues, and treatment considerations. Current Psychiatry Reports, 21(5), 34. 10.1007/s11920-019-1020-5 30903299

[jcv212259-bib-0003] Arnett, A. B. , Pennington, B. F. , Friend, A. , Willcutt, E. G. , Byrne, B. , Samuelsson, S. , & Olson, R. K. (2013). The SWAN captures variance at the negative and positive ends of the ADHD symptom dimension. Journal of Attention Disorders, 17(2), 152–162. 10.1177/1087054711427399 22173148 PMC3330134

[jcv212259-bib-0004] Ashinoff, B. K. , & Abu‐Akel, A. (2021). Hyperfocus: The forgotten Frontier of attention. Psychological Research, 85, 1–19. 10.1007/s00426-019-01245-8 31541305 PMC7851038

[jcv212259-bib-0005] Asparouhov, T. , & Muthén, B. O. (2020). Auxiliary variables in mixture modeling: Using the BCH method in Mplus to estimate a distal outcome model and an arbitrary secondary model. Mplus Web Notes.

[jcv212259-bib-0006] Bagozzi, R. P. , & Yi, Y. (2012). Specification, evaluation, and interpretation of structural equation models. Journal of the Academy of Marketing Science, 40(1), 8–34. 10.1007/s11747-011-0278-x

[jcv212259-bib-0007] Bauer, D. J. , & Curran, P. J. (2004). The integration of continuous and discrete latent variable models: Potential problems and promising opportunities. Psychological Methods, 9(1), 3–29. 10.1037/1082-989x.9.1.3 15053717

[jcv212259-bib-0008] Benjamini, Y. , & Hochberg, Y. (1995). Controlling the false discovery rate: A practical and powerful approach to multiple testing. Journal of the Royal Statistical Society: Series B, 57(1), 289–300. 10.1111/j.2517-6161.1995.tb02031.x

[jcv212259-bib-0009] Bergold, J. , & Thomas, S. (2012). Participatory research methods: A methodological approach in motion. Historical Social Research / Historische Sozialforschung, 37, 191–222.

[jcv212259-bib-0010] Bondü, R. , & Esser, G. (2015). Justice and rejection sensitivity in children and adolescents with ADHD symptoms (Vol. 24, pp. 185–198). European Child & Adolescent Psychiatry. 10.1007/s00787-014-0560-9 24878677

[jcv212259-bib-0011] Bottema‐Beutel, K. , Kapp, S. K. , Lester, J. N. , Sasson, N. J. , & Hand, B. N. (2021). Avoiding ableist language: Suggestions for autism researchers. Autism Adulthood, 3(1), 18–29. 10.1089/aut.2020.0014 36601265 PMC8992888

[jcv212259-bib-0012] Brown, M. B. , & Forsythe, A. B. (1974). Robust tests for the equality of variances. Journal of the American Statistical Association, 69(346), 364–367. 10.2307/2285659

[jcv212259-bib-0013] Carpenter, K. L. H. , Davis, N. O. , Spanos, M. , Sabatos‐Devito, M. , Aiello, R. , Baranek, G. T. , Compton, S. N. , Egger, H. L. , Franz, L. , Kim, S. J. , King, B. H. , Kolevzon, A. , Mcdougle, C. J. , Sanders, K. , Veenstra‐Vanderweele, J. , Sikich, L. , Kollins, S. H. , & Dawson, G. (2022). Adaptive behavior in young autistic children: Associations with irritability and ADHD symptoms. Journal of Autism and Developmental Disorders. 10.1007/s10803-022-05753-2 PMC10090229 36222990

[jcv212259-bib-0014] Chau, T. , Tiego, J. , Brown, L. , Mellahn, O. , Johnson, B. P. , Arnatkeviciute, A. , Fulcher, B. D. , Matthews, N. , & Bellgrove, M. A. (2023). The distribution of parent‐reported autistic and ADHD traits in children with and without an ADHD diagnosis. PsyArXiv. 10.1002/jcv2.12223 PMC11143953 38827983

[jcv212259-bib-0015] Clark, S. L. , Muthén, B. , Kaprio, J. , D'onofrio, B. M. , Viken, R. , & Rose, R. J. (2013). Models and strategies for factor mixture analysis: An example concerning the structure underlying psychological disorders. Structural Equation Modeling, 20(4), 1070–5511. 10.1080/10705511.2013.824786 PMC3844130 24302849

[jcv212259-bib-0016] Clark, S. L. , & Muthén, B. O. (2009). Relating latent class analysis results to variables not included in the analysis.

[jcv212259-bib-0017] Constantino, J. N. , & Gruber, C. P. (2012). Social responsiveness scale (2nd ed.). Western Psychological Services. *(SRS‐2)*.

[jcv212259-bib-0018] Constantino, J. N. , & Todd, R. D. (2003). Autistic traits in the general population: A twin study. Archives of General Psychiatry, 60(5), 524–530. 10.1001/archpsyc.60.5.524 12742874

[jcv212259-bib-0019] Deserno, M. K. , Bathelt, J. , Groenman, A. P. , & Geurts, H. M. (2022). Probing the overarching continuum theory: Data‐driven phenotypic clustering of children with ASD or ADHD. European Child & Adolescent Psychiatry. 10.1007/s00787-022-01986-9 PMC10533623 35687205

[jcv212259-bib-0020] Dupuis, A. , Mudiyanselage, P. , Burton, C. L. , Arnold, P. D. , Crosbie, J. , & Schachar, R. J. (2022). Hyperfocus or flow? Attentional strengths in autism spectrum disorder. Frontiers in Psychiatry, 13. 10.3389/fpsyt.2022.886692 PMC9579965 36276327

[jcv212259-bib-0021] Duvekot, J. , Van Der Ende, J. , Verhulst, F. C. , Slappendel, G. , Van Daalen, E. , Maras, A. , & Greaves‐Lord, K. (2017). Factors influencing the probability of a diagnosis of autism spectrum disorder in girls versus boys. Autism, 21(6), 646–658. 10.1177/1362361316672178 27940569

[jcv212259-bib-0022] Enders, C. K. (2010). Applied missing data analysis. The Guilford Press.

[jcv212259-bib-0023] Frazier, T. W. , Ratliff, K. R. , Gruber, C. , Zhang, Y. , Law, P. A. , & Constantino, J. N. (2014). Confirmatory factor analytic structure and measurement invariance of quantitative autistic traits measured by the Social Responsiveness Scale‐2. Autism, 18(1), 31–44. 10.1177/1362361313500382 24019124

[jcv212259-bib-0024] Games, P. A. , Keselman, H. J. , & Clinch, J. J. (1979). Tests for homogeneity of variance in factorial designs. Psychological Bulletin, 86(5), 978–984. 10.1037//0033-2909.86.5.978

[jcv212259-bib-0025] Ghanizadeh, A. (2012). Co‐morbidity and factor analysis on attention deficit hyperactivity disorder and autism spectrum disorder DSM‐IV‐derived items. Journal of Research in Medical Sciences, 17, 368–372. 23267399 PMC3526131

[jcv212259-bib-0026] Greenbaum, P. , Boca, F. , Darkes, J. , Wang, C.‐P. , & Goldman, M. (2005). Variation in the drinking trajectories of freshmen college students. Journal of Consulting and Clinical Psychology, 73(2), 229–238. 10.1037/0022-006x.73.2.229 15796630

[jcv212259-bib-0027] Harkins, C. , & Mazurek, M. O. (2023). The impact of Co‐occurring ADHD on social competence intervention outcomes in youth with autism spectrum disorder. Journal of Autism and Developmental Disorders. 10.1007/s10803-023-05987-8 PMC10624644 37142907

[jcv212259-bib-0028] Hong, J. S. , Singh, V. , & Kalb, L. (2021). Attention deficit hyperactivity disorder symptoms in young children with autism spectrum disorder. Autism Research, 14(1), 182–192. 10.1002/aur.2414 33073542

[jcv212259-bib-0029] Hoogman, M. , Van Rooij, D. , Klein, M. , Boedhoe, P. , Ilioska, I. , Li, T. , Patel, Y. , Postema, M. C. , Zhang‐James, Y. , Anagnostou, E. , Arango, C. , Auzias, G. , Banaschewski, T. , Bau, C. H. D. , Behrmann, M. , Bellgrove, M. A. , Brandeis, D. , Brem, S. , Busatto, G. F. , … Franke, B. (2022). Consortium neuroscience of attention deficit/hyperactivity disorder and autism spectrum disorder: The ENIGMA adventure. Human Brain Mapping, 43(1), 37–55. 10.1002/hbm.25029 32420680 PMC8675410

[jcv212259-bib-0030] Jeffreys, H. (1961). Theory of probability. Oxford University Press.

[jcv212259-bib-0031] Jeste, S. S. , & Geschwind, D. H. (2014). Disentangling the heterogeneity of autism spectrum disorder through genetic findings. Nature Reviews Neurology, 10(2), 74–81. 10.1038/nrneurol.2013.278 24468882 PMC4125617

[jcv212259-bib-0032] Karalunas, S. L. , & Nigg, J. T. (2020). Heterogeneity and subtyping in attention‐deficit/hyperactivity disorder‐considerations for emerging research using person‐centered computational approaches. Biological Psychiatry, 88(1), 103–110. 10.1016/j.biopsych.2019.11.002 31924323 PMC7210094

[jcv212259-bib-0033] Kass, R. E. , & Raftery, A. E. (1995). Bayes factors. Journal of the American Statistical Association, 90(430), 773–795. 10.2307/2291091

[jcv212259-bib-0034] Kline, R. B. (2015). Principles and practice of structural equation modeling. Guilford Press.

[jcv212259-bib-0035] Knott, R. , Johnson, B. P. , Tiego, J. , Mellahn, O. , Finlay, A. , Kallady, K. , Kouspos, M. , Mohanakumar Sindhu, V. P. , Hawi, Z. , Arnatkeviciute, A. , Chau, T. , Maron, D. , Mercieca, E.‐C. , Furley, K. , Harris, K. , Williams, K. , Ure, A. , Fornito, A. , Gray, K. , … Bellgrove, M. A. (2021). The Monash autism‐ADHD genetics and neurodevelopment (MAGNET) project design and methodologies: A dimensional approach to understanding neurobiological and genetic aetiology. Molecular Autism, 12(1), 55. 10.1186/s13229-021-00457-3 34353377 PMC8340366

[jcv212259-bib-0036] Knott, R. , Mellahn, O. , Tiego, J. , Kallady, K. , Brown, L. , Coghill, D. , Williams, K. , Bellgrove, M. A. , & Johnson, B. P. (2024). Age at diagnosis and diagnostic delay across attention‐deficit hyperactivity and autism spectrums. Australian & New Zealand Journal of Psychiatry, 58(2),142–151. 10.1177/00048674231206997 37885260 PMC10838471

[jcv212259-bib-0037] Krakowski, A. D. , Szatmari, P. , Crosbie, J. , Schachar, R. , Duku, E. , Georgiades, S. , & Anagnostou, E. (2021). Latent structure of combined autistic and ADHD symptoms in clinical and general population samples: A scoping review. Frontiers in Psychiatry, 12. 10.3389/fpsyt.2021.654120 PMC8721217 34987421

[jcv212259-bib-0038] Lee, D. O. , & Ousley, O. Y. (2006). Attention‐deficit hyperactivity disorder symptoms in a clinic sample of children and adolescents with pervasive developmental disorders. Journal of Child and Adolescent Psychopharmacology, 16(6), 737–746. 10.1089/cap.2006.16.737 17201617

[jcv212259-bib-0039] Lee, W.‐Y. , Cho, S.‐J. , & Sterba, S. K. (2018). Ignoring a multilevel structure in mixture item response models: Impact on parameter recovery and model selection. Applied Psychological Measurement, 42(2), 136–154. 10.1177/0146621617711999 29882542 PMC5978650

[jcv212259-bib-0040] Leyfer, O. T. , Folstein, S. E. , Bacalman, S. , Davis, N. O. , Dinh, E. , Morgan, J. , Tager‐Flusberg, H. , & Lainhart, J. E. (2006). Comorbid psychiatric disorders in children with autism: Interview development and rates of disorders. Journal of Autism and Developmental Disorders, 36(7), 849–861. 10.1007/s10803-006-0123-0 16845581

[jcv212259-bib-0041] Little, L. M. , Wallisch, A. , Salley, B. , & Jamison, R. (2017). Do early caregiver concerns differ for girls with autism spectrum disorders? Autism, 21(6), 728–732. 10.1177/1362361316664188 27542396 PMC5532076

[jcv212259-bib-0042] Little, R. J. A. (1988). A test of missing completely at random for multivariate data with missing values. Journal of the American Statistical Association, 83(404), 1198–1202. 10.2307/2290157

[jcv212259-bib-0043] Liu, Y. , Wang, L. , Xie, S. , Pan, S. , Zhao, J. , Zou, M. , & Sun, C. (2021). Attention deficit/hyperactivity disorder symptoms impair adaptive and social function in children with autism spectrum disorder. Frontiers in Psychiatry, 12. 10.3389/fpsyt.2021.654485 PMC8727694 35002788

[jcv212259-bib-0044] Lo, Y. , Mendell, N. R. , & Rubin, D. B. (2001). Testing the number of components in a normal mixture. Biometrika, 88(3), 767–778. 10.1093/biomet/88.3.767

[jcv212259-bib-0045] Lubke, G. , & Neale, M. C. (2006). Distinguishing between latent classes and continuous factors: Resolution by maximum likelihood? Multivariate Behavioral Research, 41(4), 499–532. 10.1207/s15327906mbr4104_4 26794916

[jcv212259-bib-0046] Mclachlan, G. J. (1987). On bootstrapping the likelihood ratio test statistic for the number of components in a normal mixture. Journal of the Royal Statistical Society: Series C (Applied Statistics), 36(3), 318–324. 10.2307/2347790

[jcv212259-bib-0047] Mcquaid, G. A. , Lee, N. R. , & Wallace, G. L. (2022). Camouflaging in autism spectrum disorder: Examining the roles of sex, gender identity, and diagnostic timing. Autism, 26(2), 552–559. 10.1177/13623613211042131 34420418

[jcv212259-bib-0048] Mohamadesmaiel, E. , & Alipour, A. (2002). A preliminary study on the reliability, validity and cut off points of the disorders of children symptom inventory‐4 (CSI‐4). Journal of Exceptional Children, 2, 239–254.

[jcv212259-bib-0049] Muthén, L. K. , & Muthén, B. O. (2017). Mplus: Statistical analysis with latent variables: User's guide (version 8). Authors.

[jcv212259-bib-0050] Nylund, K. L. , Asparouhov, T. , & Muthén, B. O. (2007). Deciding on the number of classes in latent class analysis and growth mixture modeling: A Monte Carlo simulation study. Structural Equation Modeling: A Multidisciplinary Journal, 14(4), 535–569. 10.1080/10705510701575396

[jcv212259-bib-0051] Schaaf, R. C. , Mailloux, Z. , Ridgway, E. , Berruti, A. S. , Dumont, R. L. , Jones, E. A. , Leiby, B. E. , Sancimino, C. , Yi, M. , & Molholm, S. (2022). Sensory phenotypes in autism: Making a case for the inclusion of sensory integration functions. Journal of Autism and Developmental Disorders, 53(12), 4759–4771. 10.1007/s10803-022-05763-0 36167886

[jcv212259-bib-0052] Schachar, R. J. , Dupuis, A. , Arnold, P. D. , Anagnostou, E. , Kelley, E. , Georgiades, S. , Nicolson, R. , Townes, P. , Burton, C. L. , & Crosbie, J. (2023). Autism spectrum disorder and attention‐deficit/hyperactivity disorder: Shared or unique neurocognitive profiles? Research on Child and Adolescent Psychopathology, 51(1), 17–31. 10.1007/s10802-022-00958-6 36006496 PMC9763138

[jcv212259-bib-0053] Simonoff, E. , Pickles, A. , Charman, T. , Chandler, S. , Loucas, T. , & Baird, G. (2008). Psychiatric disorders in children with autism spectrum disorders: Prevalence, comorbidity, and associated factors in a population‐derived sample. Journal of the American Academy of Child & Adolescent Psychiatry, 47(8), 921–929. 10.1097/chi.0b013e318179964f 18645422

[jcv212259-bib-0054] Sinzig, J. , Walter, D. , & Doepfner, M. (2009). Attention deficit/hyperactivity disorder in children and adolescents with autism spectrum disorder: Symptom or syndrome? Journal of Attention Disorders, 13(2), 117–126. 10.1177/1087054708326261 19380514

[jcv212259-bib-0055] Swanson, J. , Posner, M. , Fusella, J. , Wasdell, M. , Sommer, T. , & Fan, J. (2001). Genes and attention deficit hyperactivity disorder. Current Psychiatry Reports, 3(2), 92–100. 10.1007/s11920-001-0005-2 11276403

[jcv212259-bib-0056] Tabachnick, B. G. , & Fidell, L. S. (2013). Using multivariate statistics. Pearson Education.

[jcv212259-bib-0057] Van Buuren, S. (2007). Multiple imputation of discrete and continuous data by fully conditional specification. Statistical Methods in Medical Research, 16(3), 219–242. 10.1177/0962280206074463 17621469

[jcv212259-bib-0058] Van Der Meer, J. M. , Oerlemans, A. M. , Van Steijn, D. J. , Lappenschaar, M. G. , De Sonneville, L. M. , Buitelaar, J. K. , & Rommelse, N. N. (2012). Are autism spectrum disorder and attention‐deficit/hyperactivity disorder different manifestations of one overarching disorder? Cognitive and symptom evidence from a clinical and population‐based sample. Journal of the American Academy of Child and Adolescent Psychiatry, 51(11), 1160–1172e1163. 10.1016/j.jaac.2012.08.024 23101742

[jcv212259-bib-0059] Van Der Meer, J. M. J. , Lappenschaar, M. G. A. , Hartman, C. A. , Greven, C. U. , Buitelaar, J. K. , & Rommelse, N. N. J. (2017). Homogeneous combinations of ASD‐ADHD traits and their cognitive and behavioral correlates in a population‐based sample. Journal of Attention Disorders, 21(9), 753–763. 10.1177/1087054714533194 24819924

[jcv212259-bib-0060] Wechsler, D. (2011). Wechsler abbreviated scale of intelligence (2nd ed.). NCS Pearson. *(WASI‐II)*.

[jcv212259-bib-0061] Wechsler, D. (2012). Wechsler Preschool and primary scale of intelligence (4th ed.). The Psychological Corporation.

[jcv212259-bib-0062] Wechsler, D. (2014). Wechsler intelligence scale for children (5th ed.). Pearson.

[jcv212259-bib-0063] Yerys, B. E. , Bertollo, J. R. , Pandey, J. , Guy, L. , & Schultz, R. T. (2019). Attention‐deficit/hyperactivity disorder symptoms are associated with lower adaptive behavior skills in children with autism. Journal of the American Academy of Child & Adolescent Psychiatry, 58(5), 525–533.e523. 10.1016/j.jaac.2018.08.017 31029198

