# Supplementary material for: The distribution of parent‐reported autistic and subclinical ADHD traits in children with and without an autism diagnosis
Source: JCPP Adv. 2024 Jun 28;5(1):e12259. doi: 10.1002/jcv2.12259 (PMC11889647; doi:10.1002/jcv2.12259)
Supplement: Supplementary file 1 — Supporting Information S1 [file JCV2-5-e12259-s001.docx]

**Supplementary Materials**

Appendix S1.

*Discriminant Validity*

Discriminant validity was analysed through (1) evaluation of the 95% confidence interval around the factor intercorrelation (φ), (2) a comparison of the average variance extracted with the squared factor intercorrelation (Hair, 2014; Fornell & Larcker, 1981), and (3) a comparison of a statistically nested one-factor model to the two-factor model using the Satorra-Bentler scaled difference chi-square statistic (TRd; Satorra & Bentler, 2010).

Appendix S2.

*Factor Mixture Modelling Variations*

These variations differ by their restrictions on the invariance of the following parameters across classes: factor loadings (invariant across FMM-1 through FMM-3), item thresholds (invariant across FMM-1 and FMM-2), and the factor covariance matrix (invariant in FMM-1 only). Factor means in each class are freely estimated for FMM-1 and FMM-2. Estimations commenced with the most restrictive model (i.e., FMM-1) and progressed sequentially through to the least restrictive model (i.e., FMM-4; Clark et al., 2013).

Appendix S3.

*Further Details on Class Assignment*

Class assignment of participants in mixture modelling is probabilistic. The quality of class separation is indicated by entropy (*E*), which ranges between 0.00 – 1.00, with higher values indicative of clearer class separation (Clark & Muthén, 2009). Participants can be assigned to a specific class based on the highest posterior probability. Results can then be compared across classes. When class separation is high (Entropy > .80), such that class membership can be used as a discrete categorical variable, this classify-analyse approach can be implemented (Clark & Muthén, 2009).

Appendix S4.

*Undertaking the Analyses on Data from Participants Aged 5-17 Years*

Tables AS4.1 and AS4.2 display the results from confirmatory factor analyses, latent profile analyses, and factor mixture modelling for participants aged 5-17 years in the MAGNET and HBN samples respectively.

Table AS4.1.

*Discovery Sample: Factor Analysis, Latent Profile Analysis, and Factor Mixture Modelling Results for Participants Aged 5-17 years.*

| Model | Log likelihood | Entropy | AIC | BIC | VLMR *p* |
| --- | --- | --- | --- | --- | --- |
| *Common Factor Model* | | | | | |
| 1 factor | -2527.323 | - | 5096.647 | 5157.026 | - |
| 1 factor (Nested) | -2531.401 | - | 5102.802 | 5160.306 | - |
| *Confirmatory Factor Analysis* | | | | | |
| 2 factor | -2508.631 | - | 5059.262 | 5119.641 | - |
| 2 factor (modified) | -2503.275 | - | 5056.550 | 5128.430 |  |
| *Latent Profile Analysis* | | | | | |
| *Equal Variances Across Classes* | | |  |  |  |
| 1 class | -2978.706 | - | 5985.412 | 6042.664 | - |
| 2 classes | -2667.823 | .972 | 5379.646 | 5442.901 | < .001 |
| 3 classes | -2567.556 | .954 | 5195.112 | 5281.368 | .016 |
| 4 classes | -2522.355 | .916 | 5120.709 | 5229.967 | .290 |
| 5 classes | -2492.301 | .929 | 5076.601 | 5208.860 | .313 |
| 6 classes | -2471.777 | .934 | 5051.555 | 5206.815 | .427 |
| *Freely Estimated Variances Across Classes* | | | |  |  |
| 1 class | -2978.706 | - | 5985.412 | 6042.664 | - |
| 2 classes | -2651.381 | .963 | 5360.762 | 5444.143 | < .001 |
| 3 classes | -2528.735 | .942 | 5145.469 | 5271.978 | .006 |
| 4 classes | -2464.534 | .964 | 5047.068 | 5216.705 | .203 |
| 5 classes^ |  |  |  |  |  |
| 6 classes^ |  |  |  |  |  |
| *Factor Mixture Modelling* | | | | | |
| *1 factor, 2 classes* | |  |  |  |  |
| FMM-1 | -2667.823 | .972 | 5379.646 | 5442.901 | < .001 |
| FMM-2 | -2508.659 | .862 | 5063.318 | 5129.448 | .012 |
| FMM-3 | -2492.792 | .770 | 5043.585 | 5126.965 | .352 |
| FMM-4 | -2484.451 | .911 | 5038.901 | 5139.533 | .588 |
| *1 factor, 3 classes* | |  |  |  |  |
| FMM-1 | -2572.790 | .952 | 5193.580 | 5262.585 | .013 |
| FMM-2 | -2504.374 | .828 | 5058.747 | 5130.627 | .364 |
| FMM-3 | -2471.059 | .935 | 5016.118 | 5122.500 | .454 |
| FMM-4 | -2440.774 | .910 | 4979.549 | 5120.433 | .020 |
| *2 factors, 2 classes* | |  |  |  |  |
| FMM-1^ |  |  |  |  |  |
| FMM-2 | -2479.725 | .717 | 5015.450 | 5095.955 | .173 |
| FMM-3 | -2470.475 | .907 | 5004.951 | 5096.957 | .284 |
| FMM-4^ |  |  |  |  |  |
| *2 factors, 3 classes* | |  |  |  |  |
| **FMM-1** | **-2478.582** | **.832** | **5011.165** | **5003.397** | **.513** |
| FMM-2^ |  |  |  |  |  |
| FMM-3 | 2443.522 | .959 | 4973.043 | 5096.677 | .378 |
| FMM-4^ |  |  |  |  |  |

*Note*. **Bold typeface** = best fitting model; # = Loglikehood was not replicated; ^ = Model misspecified; AIC = Akaike Information Criterion; BIC = Bayesian Information Criterion; VLMR*p* = Vuong-Lo-Mendell-Rubin *p*-value; BLRT*p* = Bootstrapped Likelihood Ratio Test *p*-value; *p* = probability value of the test statistic; FMM = factor mixture model.

Table AS4.2.

*Replication Sample: Factor Analysis, Latent Profile Analysis, and Factor Mixture Modelling Results for Participants Aged 5-17 years.*

| Model | Log likelihood | Entropy | AIC | BIC | VLMR *p* | BLRT*p* | |
| --- | --- | --- | --- | --- | --- | --- | --- |
| *Common Factor Model* | | | | | | | |
| 1 factor | -8184.338 | - | 16410.676 | 16495.321 | - | - | |
| 1 factor (nested) | -8095.221 | - | 16238.441 | 16335.178 | - | - | |
| *Confirmatory Factor Analysis* | | | | | | | |
| 2 factor | -8098.290 | - | 16238.580 | 16323.225 | - | - | |
| 2 factor (modified) | -8063.756 | - | 16177.512 | 16278.279 | - | - | |
| *Latent Profile Analysis* | | | | | | | |
| *Equal Variances Across Classes* | | |  |  |  |  | |
| 1 class | -9133.319 | - | 18294.638 | 18351.067 | - | - |  |
| 2 classes | -8543.032 | .947 | 17130.065 | 17218.740 | < .001 | < .001 |  |
| 3 classes | -8298.991 | .889 | 16657.982 | 16778.903 | .426 | < .001 |  |
| 4 classes | -8159.189 | .888 | 16394.378 | 16547.544 | .160 | < .001 |  |
| 5 classes | -8068.719 | .881 | 16229.437 | 16414.849 | .060 | < .001 |  |
| 6 classes | -8007.373 | .894 | 16122.747 | 16340.404 | .024 | < .001 |  |
| *Freely Estimated Variances Across Classes* | | | |  |  |  |  |
| 1 class | -9133.319 | - | 18294.638 | 18351.067 | - | - |  |
| 2 classes | -8374.251 | .896 | 16806.503 | 16923.392 | < .001 | < .001 |  |
| 3 classes | -8069.866 | .903 | 16227.732 | 16405.082 | < .001 | < .001 |  |
| 4 classes | -7939.715 | .895 | 15997.430 | 16235.241 | .003 | < .001 |  |
| 5 classes | -7842.925 | .893 | 15833.850 | 16132.121 | .011 | < .001 |  |
| 6 classes | -7764.996 | .903 | 15707.991 | 16066.722 | .042 | < .001 |  |
| *Factor Mixture Modelling* | | | | | | |  |
| *1 factor, 2 classes* | |  |  |  |  |  |  |
| FMM-1 | -8543.033 | .947 | 17130.065 | 17218.740 | < .001 | < .001 |  |
| FMM-2 | -8137.684 | .876 | 16321.368 | 16414.074 | < .001 | < .001 |  |
| FMM-3 | -8029.368 | .887 | 16116.736 | 16233.626 | < .001 | < .001 |  |
| FMM-4 | -8004.141 | .866 | 16078.282 | 16219.356 | < .001 | < .001 |  |
| *1 factor, 3 classes* | |  |  |  |  |  |  |
| FMM-1 | -8316.314 | .894 | 16680.628 | 16777.364 | .158 | 1.000 |  |
| FMM-2 | -8126.746 | .831 | 16303.493 | 16404.260 | .03 | 1.000 |  |
| FMM-3 | -7934.666 | .920 | 16943.333 | 16092.468 | < .001 | < .001 |  |
| FMM-4 | -7919.930 | .905 | 15937.859 | 16135.363 | .155 | < .001 |  |
| *1 factor, 4 classes* | |  |  |  |  |  |  |
| FMM-1 | -8199.504 | .876 | 16451.008 | 16555.806 | .534 | 1.000 |  |
| FMM-2 | -8124.127 | .726 | 16302.253 | 16411.082 | .526 | 1.000 |  |
| FMM-3 | -7896.002 | .928 | 15882.004 | 16063.385 | .262 | < .001 |  |
| FMM-4^ |  |  |  |  |  |  |  |
| *1 factor, 5 classes* | |  |  |  |  |  |  |
| FMM-1 | -8150.449 | .866 | 16356.897 | 16469.757 | .007 | .500 |  |
| FMM-2 | -8120.535 | .775 | 16299.070 | 16415.960 | .324 | 1.000 |  |
| FMM-3 | -7870.354 | .923 | 15846.707 | 16060.334 | .178 | < .001 |  |
| FMM-4^ |  |  |  |  |  |  |  |
| *1 factor, 6 classes* | |  |  |  |  |  |  |
| FMM-1 | -8130.145 | .875 | 16320.291 | 16441.212 | .129 | .667 |  |
| FMM-2^ |  |  |  |  |  |  |  |
| FMM-3 | -7851.031 | .916 | 15824.062 | 16069.934 | .495 | < .001 |  |
| FMM-4^ |  |  |  |  |  |  |  |
| *2 factors, 2 classes* | |  |  |  |  |  |  |
| FMM-1% |  |  |  |  |  |  |  |
| FMM-2 | -8006.029 | .686 | 16068.058 | 16180.917 | .087 | < .001 |  |
| FMM-3 | -7974.251 | .754 | 16014.502 | 16147.515 | < .001 | < .001 |  |
| FMM-4^ |  |  |  |  |  |  |  |
| *2 factors, 3 classes* | |  |  |  |  |  |  |
| FMM-1^ |  |  |  |  |  |  |  |
| FMM-2^ |  |  |  |  |  |  |  |
| **FMM-3** | **-7899.549** | **.925** | **15887.098** | **16064.448** | **< .001** | **< .001** |  |
| FMM-4^ |  |  |  |  |  |  |  |
| *2 factors, 4 classes* | |  |  |  |  |  |  |
| FMM-1^ |  |  |  |  |  |  |  |
| FMM-2^ |  |  |  |  |  |  |  |
| FMM-3 | -7859.852 | .860 | 16829.703 | 16051.391 | .266 | < .001 |  |
| FMM-4^ |  |  |  |  |  |  |  |
| *2 factors, 5 classes* | |  |  |  |  |  |  |
| FMM-1^ |  |  |  |  |  |  |  |
| FMM-2^ |  |  |  |  |  |  |  |
| FMM-3^ |  |  |  |  |  |  |  |
| FMM-4^ |  |  |  |  |  |  |  |
| *2 factors, 6 classes* | |  |  |  |  |  |  |
| FMM-1^ |  |  |  |  |  |  |  |
| FMM-2^ |  |  |  |  |  |  |  |
| FMM-3^ |  |  |  |  |  |  |  |
| FMM-4^ |  |  |  |  |  |  |  |

*Note*. **Bold typeface** = best fitting model; # = Loglikehood was not replicated; ^ = Model misspecified; AIC = Akaike Information Criterion; BIC = Bayesian Information Criterion; VLMR*p* = Vuong-Lo-Mendell-Rubin *p*-value; BLRT*p* = Bootstrapped Likelihood Ratio Test *p*-value; *p* = probability value of the test statistic; FMM = factor mixture model.

Table S1.

*Discovery Sample: Descriptive Statistics for Continuous Variables*

|  | *n* | Minimum | Maximum | *M* | *SD* | Skewness (*SE*) | Kurtosis (*SE*) |
| --- | --- | --- | --- | --- | --- | --- | --- |
| Age | 164 | 4 | 17 | 8.64 | 2.95 | .420 (.190) | - .391 (.377) |
| FSIQ | 151 | 76 | 141 | 105.57 | 12.67 | .111 (.197) | - .405 (.392) |
| SRS_Aware | 143 | 32 | 90 | 57.99 | 14.57 | .404 (.203) | - .557 (.403) |
| SRS_Cog | 143 | 39 | 90 | 55.58 | 14.04 | .690 (.203) | - .624 (.403) |
| SRS_Comm | 143 | 38 | 90 | 56.31 | 13.95 | .764 (.203) | - .409 (.403) |
| SRS_Motiv | 143 | 38 | 90 | 55.10 | 13.31 | .839 (.203) | - .166 (.403) |
| SRS_RRB | 143 | 41 | 90 | 55.78 | 14.25 | 1.05 (.203) | - .006 (.403) |
| SWAN_Inatt | 110 | -2.89 | 2.89 | .127 | 1.15 | - .059 (.230) | - .118 (.457) |
| SWAN_Hyp | 110 | -3.00 | 3.00 | .004 | 1.15 | - .557 (.230) | .840 (.457) |

*Note.* *n* = number of participants with complete data available for this measure; *M* = mean; *SD* = standard deviation; FSIQ = Full-scale intelligence; SRS = Social Responsiveness Scale, 2^nd^ Edition; SWAN = Strengths and Weaknesses of ADHD and Normal Behaviour Scale; SRS_Aware = SRS-2 Social Awareness subscale; SRS_Cog = SRS-2 Social Cognition subscale; SRS_Comm = SRS-2 Social Communication subscale; SRS_Motiv = SRS-2 Social Motivation subscale; SRS_RRB = SRS-2 Restricted Interests and Repetitive Behaviour subscale; SWAN_Inatt = SWAN Inattentive summary scale; SWAN_Hyp = SWAN Hyperactivity-Impulsivity summary scale.

Table S2.

*Discovery Sample: Descriptive Statistics for Continuous Variables (Neurotypical Group)*

|  | *n* | Minimum | Maximum | *M* | *SD* | Skewness (*SE*) | Kurtosis (*SE*) |
| --- | --- | --- | --- | --- | --- | --- | --- |
| Age | 121 | 4 | 16 | 8.60 | 3.02 | .390 (.220) | - .532 (.437) |
| FSIQ | 109 | 83 | 141 | 107.66 | 12.13 | .164 (.231) | - .237 (.459) |
| SRS_Aware | 104 | 32 | 78 | 52.43 | 11.09 | .312 (.237) | - .421 (.469) |
| SRS_Cog | 104 | 39 | 80 | 49.58 | 9.73 | 1.125 (.237) | .902 (.469) |
| SRS_Comm | 104 | 38 | 74 | 50.15 | 9.04 | .948 (.237) | .101 (.469) |
| SRS_Motiv | 104 | 38 | 78 | 50.08 | 9.34 | 1.010 (.237) | .832 (.469) |
| SRS_RRB | 104 | 41 | 82 | 49.21 | 8.14 | 1.778 (.237) | 3.73 (.469) |
| SWAN_Inatt | 77 | -2.89 | 2.00 | - .33 | .93 | - .328 (.274) | .027 (.541) |
| SWAN_Hyp | 77 | -3.00 | 1.44 | - .34 | 1.01 | - 1.00 (.274) | .498 (.541) |

*Note.* *n* = number of participants with complete data available for this measure; *M* = mean; *SD* = standard deviation; FSIQ = Full-scale intelligence; SRS = Social Responsiveness Scale, 2^nd^ Edition; SWAN = Strengths and Weaknesses of ADHD and Normal Behaviour Scale; SRS_Aware = SRS-2 Social Awareness subscale; SRS_Cog = SRS-2 Social Cognition subscale; SRS_Comm = SRS-2 Social Communication subscale; SRS_Motiv = SRS-2 Social Motivation subscale; SRS_RRB = SRS-2 Restricted Interests and Repetitive Behaviour subscale; SWAN_Inatt = SWAN Inattentive summary scale; SWAN_Hyp = SWAN Hyperactivity-Impulsivity summary scale.

Table S3.

*Discovery Sample: Descriptive Statistics for Continuous Variables (Autistic Group)*

|  | *n* | Minimum | Maximum | *M* | *SD* | Skewness (*SE*) | Kurtosis (*SE*) |
| --- | --- | --- | --- | --- | --- | --- | --- |
| Age | 43 | 4 | 17 | 8.77 | 2.767 | .578 (361) | .428 (.709) |
| FSIQ | 42 | 76 | 124 | 100.14 | 12.55 | .177 (.365) | - .969 (.717) |
| SRS_Aware | 39 | 48 | 90 | 72.79 | 12.25 | - .289 (.378) | - .904 (.741) |
| SRS_Cog | 39 | 48 | 90 | 71.59 | 10.88 | - .328 (.378) | - .611 (.741) |
| SRS_Comm | 39 | 52 | 90 | 72.74 | 11.18 | - .148 (.378) | - .773 (.741) |
| SRS_Motiv | 39 | 44 | 90 | 68.51 | 13.10 | - .169 (.378) | - .994 (.741) |
| SRS_RRB | 39 | 48 | 90 | 73.28 | 12.13 | - .131 (.378) | - 1.11 (.741) |
| SWAN_Inatt | 33 | - .89 | 2.89 | 1.19 | .87 | - .240 (.409) | .003 (.798) |
| SWAN_Hyp | 33 | -3.00 | 3.00 | .81 | 1.07 | - 1.05 (.409) | 4.033 (.798) |

*Note.* *n* = number of participants with complete data available for this measure; *M* = mean; *SD* = standard deviation; FSIQ = Full-scale intelligence; SRS = Social Responsiveness Scale, 2^nd^ Edition; SWAN = Strengths and Weaknesses of ADHD and Normal Behaviour Scale; SRS_Aware = SRS-2 Social Awareness subscale; SRS_Cog = SRS-2 Social Cognition subscale; SRS_Comm = SRS-2 Social Communication subscale; SRS_Motiv = SRS-2 Social Motivation subscale; SRS_RRB = SRS-2 Restricted Interests and Repetitive Behaviour subscale; SWAN_Inatt = SWAN Inattentive summary scale; SWAN_Hyp = SWAN Hyperactivity-Impulsivity summary scale.

Table S4.

*Discovery Sample: Shapiro-Wilk (W) Test for Normality Results*

|  | *W* Statistic | *df* | *p* |
| --- | --- | --- | --- |
| Age | .963 | 164 | < .001 |
| FSIQ | .990 | 151 | .375 |
| SRS_Aware | .967 | 143 | .002 |
| SRS_Cog | .911 | 143 | < .001 |
| SRS_Comm | .913 | 143 | < .001 |
| SRS_Motiv | .915 | 143 | < .001 |
| SRS_RRB | .851 | 143 | < .001 |
| SWAN_Inatt | .991 | 110 | .668 |
| SWAN_Hyp | .945 | 110 | < .001 |

*Note.* *W* statistic = ; *df* = degress of freedom; *p* = probability value of the test statistic; FSIQ = Full-scale intelligence; SRS = Social Responsiveness Scale, 2^nd^ Edition; SWAN = Strengths and Weaknesses of ADHD and Normal Behaviour Scale; SRS_Aware = SRS-2 Social Awareness subscale; SRS_Cog = SRS-2 Social Cognition subscale; SRS_Comm = SRS-2 Social Communication subscale; SRS_Motiv = SRS-2 Social Motivation subscale; SRS_RRB = SRS-2 Restricted Interests and Repetitive Behaviour subscale; SWAN_Inatt = SWAN Inattentive summary scale; SWAN_Hyp = SWAN Hyperactivity-Impulsivity summary scale.

Figure S1.

*Discovery Sample: Confirmatory Factor Analysis Diagram (Original Model)*


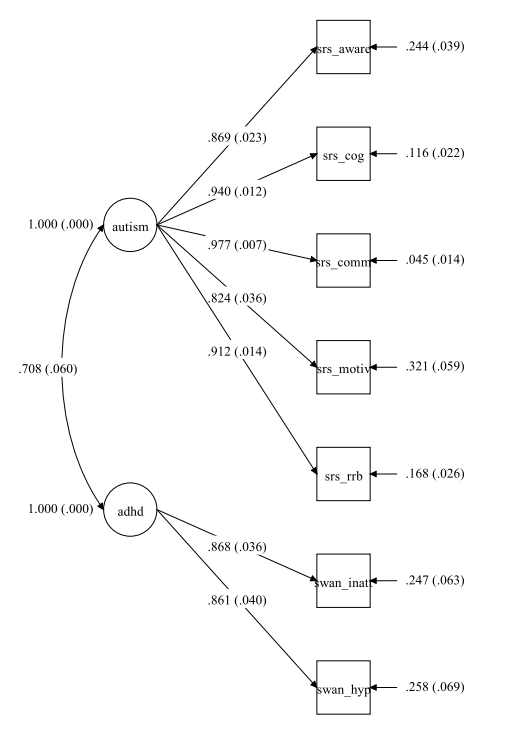


*Note.* Standardised estimates are shown with standard errors in parentheses; SRS = Social Responsiveness Scale, 2^nd^ Edition; SWAN = Strengths and Weaknesses of ADHD and Normal Behaviour Scale; ADHD = attention-deficit/hyperactivity disorder; SRS_Aware = SRS-2 Social Awareness subscale; SRS_Cog = SRS-2 Social Cognition subscale; SRS_Comm = SRS-2 Social Communication subscale; SRS_Motiv = SRS-2 Social Motivation subscale; SRS_RRB = SRS-2 Restricted Interests and Repetitive Behaviour subscale; SWAN_Inatt = SWAN Inattentive summary scale; SWAN_Hyp = SWAN Hyperactivity-Impulsivity summary scale.

Figure S2.

*Discovery Sample: Confirmatory Factor Analysis Diagram (Modified Model)*


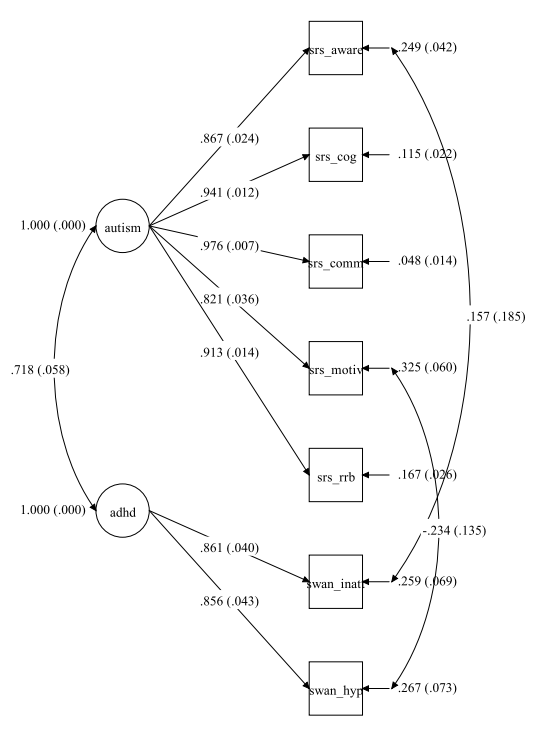


*Note.* Standardised estimates are shown with standard errors in parentheses; SRS = Social Responsiveness Scale, 2^nd^ Edition; SWAN = Strengths and Weaknesses of ADHD and Normal Behaviour Scale; ADHD = attention-deficit/hyperactivity disorder; SRS_Aware = SRS-2 Social Awareness subscale; SRS_Cog = SRS-2 Social Cognition subscale; SRS_Comm = SRS-2 Social Communication subscale; SRS_Motiv = SRS-2 Social Motivation subscale; SRS_RRB = SRS-2 Restricted Interests and Repetitive Behaviour subscale; SWAN_Inatt = SWAN Inattentive summary scale; SWAN_Hyp = SWAN Hyperactivity-Impulsivity summary scale.

Table S5.

*Discovery Sample: Confirmatory Factor Analysis Correlation Residuals – Original Model*

|  | SRS_Aware | SRS_Cog | SRS_Comm | SRS_Motiv | SRS_RRB | SWAN_Inatt | SWAN_Hyp |
| --- | --- | --- | --- | --- | --- | --- | --- |
| SRS_Aware | .000 |  |  |  |  |  |  |
| SRS_Cog | - .017 | .000 |  |  |  |  |  |
| SRS_Comm | .010 | .000 | .000 |  |  |  |  |
| SRS_Motiv | - .023 | .008 | - .005 | .000 |  |  |  |
| SRS_RRB | - .019 | .010 | - .004 | .033 | .000 |  |  |
| SWAN_Inatt | **.133** | .045 | .077 | .019 | .054 | .000 |  |
| SWAN_Hyp | .011 | - .085 | - .080 | **- .159** | - .092 | - .001 | .000 |

*Note.* **Bold typeface** = correlation residual exceeds recommended cut-off of |.10| (Kline, 2016); SRS = Social Responsiveness Scale, 2^nd^ Edition; SWAN = Strengths and Weaknesses of ADHD and Normal Behaviour Scale; SRS_Aware = SRS-2 Social Awareness subscale; SRS_Cog = SRS-2 Social Cognition subscale; SRS_Comm = SRS-2 Social Communication subscale; SRS_Motiv = SRS-2 Social Motivation subscale; SRS_RRB = SRS-2 Restricted Interests and Repetitive Behaviour subscale; SWAN_Inatt = SWAN Inattentive summary scale; SWAN_Hyp = SWAN Hyperactivity-Impulsivity summary scale.

Table S6.

*Discovery Sample: Confirmatory Factor Analysis Correlation Residuals – Modified Model*

|  | SRS_Aware | SRS_Cog | SRS_Comm | SRS_Motiv | SRS_RRB | SWAN_Inatt | SWAN_Hyp |
| --- | --- | --- | --- | --- | --- | --- | --- |
| SRS_Aware | .000 |  |  |  |  |  |  |
| SRS_Cog | - .015 | .000 |  |  |  |  |  |
| SRS_Comm | .014 | .000 | .000 |  |  |  |  |
| SRS_Motiv | - .019 | .010 | - .002 | .000 |  |  |  |
| SRS_RRB | - .017 | .009 | - .004 | .035 | .000 |  |  |
| SWAN_Inatt | .092 | .041 | .075 | .018 | .051 | .000 |  |
| SWAN_Hyp | .008 | - .089 | - .084 | - .092 | - .096 | .010 | .000 |

*Note.* **Bold typeface** = correlation residual exceeds recommended cut-off of |.10| (Kline, 2016); SRS = Social Responsiveness Scale, 2^nd^ Edition; SWAN = Strengths and Weaknesses of ADHD and Normal Behaviour Scale; SRS_Aware = SRS-2 Social Awareness subscale; SRS_Cog = SRS-2 Social Cognition subscale; SRS_Comm = SRS-2 Social Communication subscale; SRS_Motiv = SRS-2 Social Motivation subscale; SRS_RRB = SRS-2 Restricted Interests and Repetitive Behaviour subscale; SWAN_Inatt = SWAN Inattentive summary scale; SWAN_Hyp = SWAN Hyperactivity-Impulsivity summary scale.

Table S7.

*Discovery Sample: Confirmatory Factor Analysis Factor Loading Coefficients*

|  | Original model | | Modified model | |
| --- | --- | --- | --- | --- |
|  | Estimate (*S.E*.) | Standardised estimate (*S.E*.) | Estimate (*S.E*.) | Standardised estimate (*S.E*.) |
| *Autism* |  |  |  |  |
| SRS_Aware | 1.000 (.000) | .869 (.023) | 1.000 (.000) | .867 (.024) |
| SRS_Cog | 1.043 (.067) | .940 (.012) | 1.053 (.074) | .941 (.012) |
| SRS_Comm | 1.077 (.057) | .977 (.007) | 1.085 (.063) | .976 (.007) |
| SRS_Motiv | .866 (.072) | .824 (.036) | .861 (.077) | .821 (.036) |
| SRS_RRB | 1.027 (.069) | .912 (.014) | 1.037 (.076) | .913 (.014) |
| *ADHD* |  |  |  |  |
| SWAN_Inatt | 1.000 (.000) | .868 (.036) | 1.000 (.000) | .861 (.040) |
| SWAN_Hyp | .971 (.082) | .861 (.040) | .978 (.083) | .856 (.043) |
| Autism with ADHD | 8.893 (1.579) | .708 (.060) | 8.786 (1.622) | .718 (.058) |

*Note*. All factor loadings were significant (*p* < .001); *S.E*. = standard error; SRS = Social Responsiveness Scale, 2^nd^ Edition; SWAN = Strengths and Weaknesses of ADHD and Normal Behaviour Scale; SRS_Aware = SRS-2 Social Awareness subscale; SRS_Cog = SRS-2 Social Cognition subscale; SRS_Comm = SRS-2 Social Communication subscale; SRS_Motiv = SRS-2 Social Motivation subscale; SRS_RRB = SRS-2 Restricted Interests and Repetitive Behaviour subscale; SWAN_Inatt = SWAN Inattentive summary scale; SWAN_Hyp = SWAN Hyperactivity-Impulsivity summary scale.

Table S8.

*Discovery Sample – 2 factor, 3 class FMM-1: BCH Procedure Chi-square Values*

| Test | χ^2^ | *p* value |
| --- | --- | --- |
| Age – overall test | 1.736 | .420 |
| FSIQ – overall test | 17.117 | < .001 |
| Class 1 vs. 2 | .055 | .815 |
| Class 1 vs. 3 | 3.648 | .056 |
| Class 2 vs. 3 | 11.659 | .001 |

*Note*. *FMM* = factor mixture model; BCH = procedure for comparing continuous variables across latent classes that takes the probabilistic nature of class assignment into account (Asparouhov & Muthén, 2020); χ^2^ = chi square value; *p*-value = probability value of the χ^2^ statistic; FSIQ = full-scale intelligence.

Table S9.

*Discovery Sample – 2 factor, 3 class FMM-1: BCH Procedure Means and Standard Errors*

| Variable | *M* | *SE* |
| --- | --- | --- |
| *Age* |  |  |
| Class 1 | 8.184 | .646 |
| Class 2 | 9.619 | .841 |
| Class 3 | 8.459 | .362 |
| *FSIQ* |  |  |
| Class 1 | 101.160 | 4.129 |
| Class 2 | 99.904 | 2.300 |
| Class 3 | 109.333 | 1.458 |

*Note*. *FMM* = factor mixture model; BCH = procedure for comparing continuous variables across latent classes that takes the probabilistic nature of class assignment into account (Asparouhov & Muthén, 2020); χ^2^ = chi square value; *p*-value = probability value of the χ^2^ statistic; FSIQ = full-scale intelligence.

Table S10.

*Replication Sample: Descriptive Statistics for Continuous Variables (Pre-Imputation)*

|  | *n* | Minimum | Maximum | *M* | *SD* | Skewness (*SE*) | Kurtosis (*SE*) |
| --- | --- | --- | --- | --- | --- | --- | --- |
| Age | 418 | 5.097 | 18.914 | 9.9613 | 3.17325 | .608 (.119) | - .400 (.238) |
| FSIQ | 370 | 70 | 145 | 106.57 | 15.199 | - .018 (.127) | - .315 (.253) |
| SRS_Aware | 397 | 32 | 90 | 53.98 | 10.712 | .488 (.122) | .373 (.244) |
| SRS_Cog | 397 | 39 | 89 | 51.96 | 10.956 | 1.094 (.122) | .876 (.244) |
| SRS_Comm | 397 | 38 | 90 | 52.00 | 10.712 | 1.190 (.122) | 1.23 (.244) |
| SRS_Motiv | 397 | 38 | 90 | 51.32 | 10.128 | .958 (.122) | .965 (.244) |
| SRS_RRB | 397 | 41 | 90 | 51.34 | 11.227 | 1.565 (.122) | 1.95 (.244) |
| SWAN_Inatt | 405 | -3.00 | 2.889 | - .1728 | 1.1456 | - .293 (.121) | - .181 (.242) |
| SWAN_Hyp | 405 | -3.00 | 2.555 | - .2999 | 1.1448 | - .682 (.121) | .139 (.242) |

*Note.* *n* = number of participants with complete data available for this measure; *M* = mean; *SD* = standard deviation; FSIQ = Full-scale intelligence; SRS = Social Responsiveness Scale, 2^nd^ Edition; SWAN = Strengths and Weaknesses of ADHD and Normal Behaviour Scale; SRS_Aware = SRS-2 Social Awareness subscale; SRS_Cog = SRS-2 Social Cognition subscale; SRS_Comm = SRS-2 Social Communication subscale; SRS_Motiv = SRS-2 Social Motivation subscale; SRS_RRB = SRS-2 Restricted Interests and Repetitive Behaviour subscale; SWAN_Inatt = SWAN Inattentive summary scale; SWAN_Hyp = SWAN Hyperactivity-Impulsivity summary scale.

Table S11.

*Replication Sample: Descriptive Statistics for Continuous Variables (Pre-Imputation; Neurotypical Group)*

|  | *n* | Minimum | Maximum | *M* | *SD* | Skewness (*SE*) | Kurtosis (*SE*) |
| --- | --- | --- | --- | --- | --- | --- | --- |
| Age | 351 | 5.10 | 18.91 | 9.82 | 3.09 | .630 (.130) | - .307 (.260) |
| FSIQ | 311 | 70 | 145 | 107.71 | 14.490 | - .015 (.138) | - .137 (.276) |
| SRS_Aware | 333 | 32 | 87 | 51.25 | 9.074 | .196 (.134) | .080 (.266) |
| SRS_Cog | 333 | 39 | 84 | 49.05 | 8.138 | .964 (.134) | .938 (.266) |
| SRS_Comm | 333 | 38 | 87 | 49.03 | 7.853 | 1.22 (.134) | 2.29 (.266) |
| SRS_Motiv | 333 | 38 | 78 | 49.24 | 8.540 | .821 (.134) | .332 (.266) |
| SRS_RRB | 333 | 41 | 86 | 48.11 | 7.547 | 1.96 (.134) | 5.17 (.266) |
| SWAN_Inatt | 338 | -3.00 | 2.89 | - .32 | 1.10 | - .384 (.133) | - .322 (.265) |
| SWAN_Hyp | 338 | -3.00 | 2.22 | - .43 | 1.10 | - .871 (.133) | - .016 (.265) |

*Note.* *n* = number of participants with complete data available for this measure; *M* = mean; *SD* = standard deviation; FSIQ = Full-scale intelligence; SRS = Social Responsiveness Scale, 2^nd^ Edition; SWAN = Strengths and Weaknesses of ADHD and Normal Behaviour Scale; SRS_Aware = SRS-2 Social Awareness subscale; SRS_Cog = SRS-2 Social Cognition subscale; SRS_Comm = SRS-2 Social Communication subscale; SRS_Motiv = SRS-2 Social Motivation subscale; SRS_RRB = SRS-2 Restricted Interests and Repetitive Behaviour subscale; SWAN_Inatt = SWAN Inattentive summary scale; SWAN_Hyp = SWAN Hyperactivity-Impulsivity summary scale.

Table S12.

*Replication Sample: Descriptive Statistics for Continuous Variables (Pre-Imputation; Autistic Group)*

|  | *n* | Minimum | Maximum | *M* | *SD* | Skewness (*SE*) | Kurtosis (*SE*) |
| --- | --- | --- | --- | --- | --- | --- | --- |
| Age | 67 | 5.61 | 18.83 | 10.71 | 3.50 | .430 (.293) | - .839 (.578) |
| FSIQ | 59 | 75 | 139 | 100.56 | 17.428 | .331 (.311) | - .725 (.613) |
| SRS_Aware | 64 | 45 | 90 | 65.69 | 10.476 | .256 (.299) | - .551 (.590) |
| SRS_Cog | 64 | 44 | 89 | 67.08 | 11.344 | .071 (.299) | - .776 (.590) |
| SRS_Comm | 64 | 50 | 90 | 67.42 | 10.398 | .316 (.299) | - .496 (.590) |
| SRS_Motiv | 64 | 42 | 90 | 62.13 | 10.907 | .673 (.299) | .401 (.590) |
| SRS_RRB | 64 | 45 | 90 | 68.09 | 12.332 | .048 (.299) | - .914 (.590) |
| SWAN_Inatt | 67 | -2.56 | 2.78 | .55 | 1.118 | - .386 (.293) | .322 (.578) |
| SWAN_Hyp | 67 | -2.67 | 2.56 | .36 | 1.139 | - .504 (.293) | .197 (.578) |

*Note.* *n* = number of participants with complete data available for this measure; *M* = mean; *SD* = standard deviation; FSIQ = Full-scale intelligence; SRS = Social Responsiveness Scale, 2^nd^ Edition; SWAN = Strengths and Weaknesses of ADHD and Normal Behaviour Scale; SRS_Aware = SRS-2 Social Awareness subscale; SRS_Cog = SRS-2 Social Cognition subscale; SRS_Comm = SRS-2 Social Communication subscale; SRS_Motiv = SRS-2 Social Motivation subscale; SRS_RRB = SRS-2 Restricted Interests and Repetitive Behaviour subscale; SWAN_Inatt = SWAN Inattentive summary scale; SWAN_Hyp = SWAN Hyperactivity-Impulsivity summary scale.

Table S13.

*Replication Sample: Descriptive Statistics for Continuous Variables (Post-Imputation)*

|  | *n* | Minimum | Maximum | *M* | *SD* | Skewness (*SE*) | Kurtosis (*SE*) |
| --- | --- | --- | --- | --- | --- | --- | --- |
| Age | 418 | 5.10 | 18.91 | 9.9613 | 3.17325 | .608 (.119) | -.400 (.238) |
| FSIQ | 418 | 70.00 | 145.00 | 106.6470 | 14.43959 | -.032 (.119) | -.072 (.238) |
| SRS_Aware | 418 | 32.00 | 90.00 | 53.6523 | 10.51980 | .484 (.119) | .455 (.238) |
| SRS_Cog | 418 | 39.00 | 89.00 | 52.0068 | 10.77418 | 1.101 (.119) | .951 (.238) |
| SRS_Comm | 418 | 38.00 | 90.00 | 52.0363 | 10.54338 | 1.199 (.119) | 1.305 (.238) |
| SRS_Motiv | 418 | 38.00 | 90.00 | 51.3171 | 9.92686 | .974 (.119) | 1.092 (.238) |
| SRS_RRB | 418 | 41.00 | 90.00 | 51.3831 | 11.06036 | 1.570 (.119) | 2.013 (.238) |
| SWAN_Inatt | 418 | -3.00 | 2.88 | -.177 | 1.13 | -.287 (.119) | -.128 (.238) |
| SWAN_Hyp | 418 | -3.00 | 2.55 | -.307 | 1.13 | -.670 (.119) | .189 (.238) |

*Note.* *n* = number of participants with complete data available for this measure; *M* = mean; *SD* = standard deviation; FSIQ = Full-scale intelligence; SRS = Social Responsiveness Scale, 2^nd^ Edition; SWAN = Strengths and Weaknesses of ADHD and Normal Behaviour Scale; SRS_Aware = SRS-2 Social Awareness subscale; SRS_Cog = SRS-2 Social Cognition subscale; SRS_Comm = SRS-2 Social Communication subscale; SRS_Motiv = SRS-2 Social Motivation subscale; SRS_RRB = SRS-2 Restricted Interests and Repetitive Behaviour subscale; SWAN_Inatt = SWAN Inattentive summary scale; SWAN_Hyp = SWAN Hyperactivity-Impulsivity summary scale.

Table S14.

*Replication Sample: Descriptive Statistics for Continuous Variables (Post-Imputation; Neurotypical Group)*

|  | *n* | Minimum | Maximum | *M* | *SD* | Skewness (*SE*) | Kurtosis (*SE*) |
| --- | --- | --- | --- | --- | --- | --- | --- |
| Age | 351 | 5.10 | 18.91 | 9.8194 | 3.09226 | .630 (.130) | -.307 (.260) |
| FSIQ | 351 | 70.00 | 145.00 | 107.8414 | 13.73950 | -.038 (.130) | .145 (.260) |
| SRS_Aware | 351 | 32.00 | 87.00 | 51.3438 | 8.86922 | .171 (.130) | .196 (.260) |
| SRS_Cog | 351 | 39.00 | 84.00 | 49.1262 | 7.94811 | .955 (.130) | 1.069 (.260) |
| SRS_Comm | 351 | 38.00 | 87.00 | 49.0935 | 7.66584 | 1.223 (.130) | 2.489 (.260) |
| SRS_Motiv | 351 | 38.00 | 78.00 | 49.2585 | 8.33079 | .832 (.130) | .483 (.260) |
| SRS_RRB | 351 | 41.00 | 86.00 | 48.1861 | 7.37433 | 1.966 (.130) | 5.418 (.260) |
| SWAN_Inatt | 351 | -3.00 | 2.89 | -.314626 | 1.0817 | -.389 (.130) | -.257 (.260) |
| SWAN_Hyp | 351 | -3.00 | 2.22 | -.433667 | 1.0845 | -.869 (.130) | .053 (.260) |

*Note.* *n* = number of participants with complete data available for this measure; *M* = mean; *SD* = standard deviation; FSIQ = Full-scale intelligence; SRS = Social Responsiveness Scale, 2^nd^ Edition; SWAN = Strengths and Weaknesses of ADHD and Normal Behaviour Scale; SRS_Aware = SRS-2 Social Awareness subscale; SRS_Cog = SRS-2 Social Cognition subscale; SRS_Comm = SRS-2 Social Communication subscale; SRS_Motiv = SRS-2 Social Motivation subscale; SRS_RRB = SRS-2 Restricted Interests and Repetitive Behaviour subscale; SWAN_Inatt = SWAN Inattentive summary scale; SWAN_Hyp = SWAN Hyperactivity-Impulsivity summary scale.

Table S15.

*Replication Sample: Descriptive Statistics for Continuous Variables (Post-Imputation; Autistic Group)*

|  | *n* | Minimum | Maximum | *M* | *SD* | Skewness (*SE*) | Kurtosis (*SE*) |
| --- | --- | --- | --- | --- | --- | --- | --- |
| Age | 67 | 5.61 | 18.83 | 10.7047 | 3.49929 | .430 (.293) | -.839 (.578) |
| FSIQ | 67 | 75.00 | 139.00 | 100.3895 | 16.39943 | .378 (.293) | -.427 (.578) |
| SRS_Aware | 67 | 45.00 | 90.00 | 65.7458 | 10.25904 | .245 (.293) | -.462 (.578) |
| SRS_Cog | 67 | 44.00 | 89.00 | 67.0973 | 11.09622 | .067 (.293) | -.679 (.578) |
| SRS_Comm | 67 | 50.00 | 90.00 | 67.4530 | 10.16768 | .313 (.293) | -.388 (.578) |
| SRS_Motiv | 67 | 42.00 | 90.00 | 62.1017 | 10.67521 | .691 (.293) | .542 (.578) |
| SRS_RRB | 67 | 45.00 | 90.00 | 68.1319 | 12.05824 | .040 (.293) | -.819 (.578) |
| SWAN_Inatt | 67 | -2.56 | 2.78 | .5456 | 1.1176 | -.386 (.293) | .322 (.578) |
| SWAN_Hyp | 67 | -2.67 | 2.56 | .3549 | 1.1391 | -.504 (.293) | .197 (.578) |

*Note.* *n* = number of participants with complete data available for this measure; *M* = mean; *SD* = standard deviation; FSIQ = Full-scale intelligence; SRS = Social Responsiveness Scale, 2^nd^ Edition; SWAN = Strengths and Weaknesses of ADHD and Normal Behaviour Scale; SRS_Aware = SRS-2 Social Awareness subscale; SRS_Cog = SRS-2 Social Cognition subscale; SRS_Comm = SRS-2 Social Communication subscale; SRS_Motiv = SRS-2 Social Motivation subscale; SRS_RRB = SRS-2 Restricted Interests and Repetitive Behaviour subscale; SWAN_Inatt = SWAN Inattentive summary scale; SWAN_Hyp = SWAN Hyperactivity-Impulsivity summary scale.

Table S16.

*Replication Sample: Shapiro-Wilk (W) Test for Normality Results (Post-Imputation)*

|  | *W* Statistic | *df* | *p* |
| --- | --- | --- | --- |
| Age | .953 | 418 | < .001 |
| FSIQ | .991 | 418 | .014 |
| SRS_Aware | .980 | 418 | < .001 |
| SRS_Cog | .908 | 418 | < .001 |
| SRS_Comm | .903 | 418 | < .001 |
| SRS_Motiv | .934 | 418 | < .001 |
| SRS_RRB | .815 | 418 | < .001 |
| SWAN_Inatt | .979 | 418 | < .001 |
| SWAN_Hyp | .936 | 418 | < .001 |

*Note.* *W* Statistic = ; *df* = degrees of freedom; *p* = *p*-value of the *W* test statistic; FSIQ = Full scale intelligence; SRS = Social Responsiveness Scale, 2^nd^ Edition; SWAN = Strengths and Weaknesses of ADHD and Normal Behaviour Scale; SRS_Aware = SRS-2 Social Awareness subscale; SRS_Cog = SRS-2 Social Cognition subscale; SRS_Comm = SRS-2 Social Communication subscale; SRS_Motiv = SRS-2 Social Motivation subscale; SRS_RRB = SRS-2 Restricted Interests and Repetitive Behaviour subscale; SWAN_Inatt = SWAN Inattentive summary scale; SWAN_Hyp = SWAN Hyperactivity-Impulsivity summary scale.

Figure S3.

*Replication Sample: Confirmatory Factor Analysis Diagram (Original Model)*


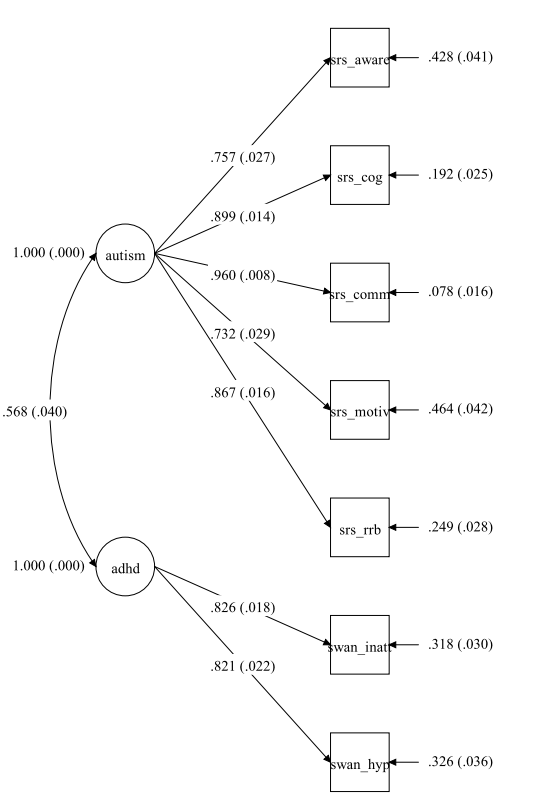


*Note.* Standardised estimates are shown with standard errors in parentheses; SRS = Social Responsiveness Scale, 2^nd^ Edition; SWAN = Strengths and Weaknesses of ADHD and Normal Behaviour Scale; ADHD = Attention-deficit/hyperactivity disorder; SRS_Aware = SRS-2 Social Awareness subscale; SRS_Cog = SRS-2 Social Cognition subscale; SRS_Comm = SRS-2 Social Communication subscale; SRS_Motiv = SRS-2 Social Motivation subscale; SRS_RRB = SRS-2 Restricted Interests and Repetitive Behaviour subscale; SWAN_Inatt = SWAN Inattentive summary scale; SWAN_Hyp = SWAN Hyperactivity-Impulsivity summary scale.

Figure S4.

*Replication Sample: Confirmatory Factor Analysis Diagram (Modified Model)*


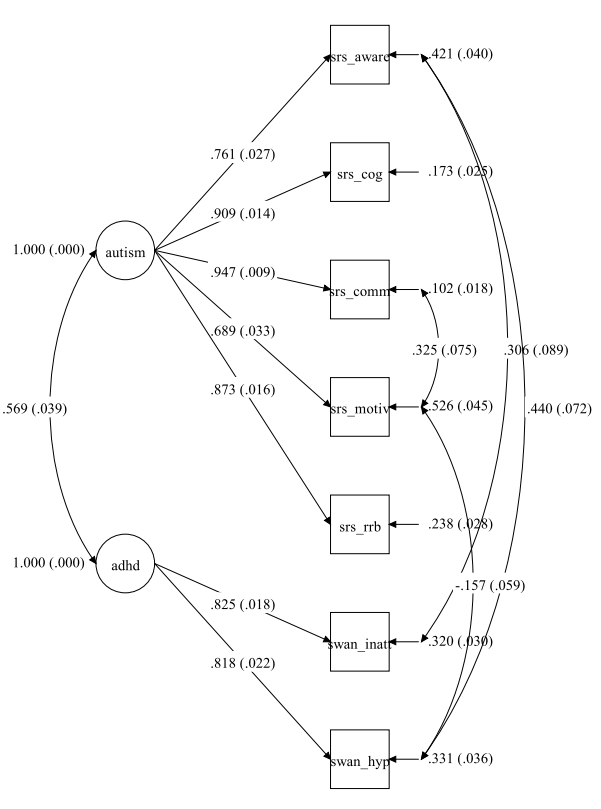


*Note.* Standardised estimates are shown with standard errors in parentheses; SRS = Social Responsiveness Scale, 2^nd^ Edition; SWAN = Strengths and Weaknesses of ADHD and Normal Behaviour Scale; ADHD = Attention-deficit/hyperactivity disorder; SRS_Aware = SRS-2 Social Awareness subscale; SRS_Cog = SRS-2 Social Cognition subscale; SRS_Comm = SRS-2 Social Communication subscale; SRS_Motiv = SRS-2 Social Motivation subscale; SRS_RRB = SRS-2 Restricted Interests and Repetitive Behaviour subscale; SWAN_Inatt = SWAN Inattentive summary scale; SWAN_Hyp = SWAN Hyperactivity-Impulsivity summary scale.

Table S17.

*Replication Sample: Confirmatory Factor Analysis Correlation Residuals – Original Model*

|  | SRS_Aware | SRS_Cog | SRS_Comm | SRS_Motiv | SRS_RRB | SWAN_Inatt | SWAN_Hyp |
| --- | --- | --- | --- | --- | --- | --- | --- |
| SRS_Aware | .000 |  |  |  |  |  |  |
| SRS_Cog | .016 | .000 |  |  |  |  |  |
| SRS_Comm | - .003 | - .005 | .000 |  |  |  |  |
| SRS_Motiv | - .046 | - .027 | .029 | .000 |  |  |  |
| SRS_RRB | - .022 | .019 | - .002 | - .019 | .000 |  |  |
| SWAN_Inatt | **.143** | .068 | .025 | - .044 | .030 | .000 |  |
| SWAN_Hyp | **.143** | - .035 | - .055 | **- .154** | - .031 | .000 | .000 |

*Note.* **Bold typeface** = correlation residual exceeds recommended cut-off of |.10| (Kline, 2016); SRS = Social Responsiveness Scale, 2^nd^ Edition; SWAN = Strengths and Weaknesses of ADHD and Normal Behaviour Scale; SRS_Aware = SRS-2 Social Awareness subscale; SRS_Cog = SRS-2 Social Cognition subscale; SRS_Comm = SRS-2 Social Communication subscale; SRS_Motiv = SRS-2 Social Motivation subscale; SRS_RRB = SRS-2 Restricted Interests and Repetitive Behaviour subscale; SWAN_Inatt = SWAN Inattentive summary scale; SWAN_Hyp = SWAN Hyperactivity-Impulsivity summary scale.

Table S18.

*Replication Sample: Confirmatory Factor Analysis Correlation Residuals – Modified Model*

|  | SRS_Aware | SRS_Cog | SRS_Comm | SRS_Motiv | SRS_RRB | SWAN_Inatt | SWAN_Hyp |
| --- | --- | --- | --- | --- | --- | --- | --- |
| SRS_Aware | .000 |  |  |  |  |  |  |
| SRS_Cog | .004 | .000 |  |  |  |  |  |
| SRS_Comm | .003 | - .004 | .000 |  |  |  |  |
| SRS_Motiv | - .016 | .004 | .004 | .000 |  |  |  |
| SRS_RRB | - .031 | .004 | .003 | .014 | .000 |  |  |
| SWAN_Inatt | .029 | .063 | .031 | - .024 | .027 | .000 |  |
| SWAN_Hyp | - .023 | - .040 | - .048 | **-** .067 | - .033 | .004 | .000 |

*Note.* **Bold typeface** = correlation residual exceeds recommended cut-off of |.10| (Kline, 2016); SRS = Social Responsiveness Scale, 2^nd^ Edition; SWAN = Strengths and Weaknesses of ADHD and Normal Behaviour Scale; SRS_Aware = SRS-2 Social Awareness subscale; SRS_Cog = SRS-2 Social Cognition subscale; SRS_Comm = SRS-2 Social Communication subscale; SRS_Motiv = SRS-2 Social Motivation subscale; SRS_RRB = SRS-2 Restricted Interests and Repetitive Behaviour subscale; SWAN_Inatt = SWAN Inattentive summary scale; SWAN_Hyp = SWAN Hyperactivity-Impulsivity summary scale.

Table S19.

*Replication Sample: Confirmatory Factor Analysis Factor Loading Coefficients*

|  | Original model | | Modified model | |
| --- | --- | --- | --- | --- |
|  | Estimate (*S.E*.) | Standardised estimate (*S.E*.) | Estimate (*S.E*.) | Standardised estimate (*S.E*.) |
| *Autism* |  |  |  |  |
| SRS_Aware | 1.000 (.000) | .757 (.027) | 1.000 (.000) | .761 (.027) |
| SRS_Cog | 1.217 (.069) | .899 (.014) | 1.220 (.070 | .909 (.014) |
| SRS_Comm | 1.272 (.074) | .960 (.008) | 1.243 (.071) | .947 (.009) |
| SRS_Motiv | .913 (.079) | .732 (.029) | .846 (.074) | .689 (.033) |
| SRS_RRB | 1.204 (.081) | .867 (.016) | 1.202 (.082) | .873 (.016) |
| *ADHD* |  |  |  |  |
| SWAN_Inatt | 1.000 (.000) | .826 (.018) | 1.000 (.000) | .825 (.018) |
| SWAN_Hyp | .980 (.049) | .821 (.022) | .974 (.049) | .818 (.022) |
| Autism with ADHD | 4.238 (.594) | .568 (.040) | 4.281 (.585) | .569 (.039) |

*Note*. All factor loadings were significant (*p* < .001); S.E. = standard error; SRS = Social Responsiveness Scale, 2^nd^ Edition; SWAN = Strengths and Weaknesses of ADHD and Normal Behaviour Scale; ADHD = attention-deficit/hyperactivity disorder; SRS_Aware = SRS-2 Social Awareness subscale; SRS_Cog = SRS-2 Social Cognition subscale; SRS_Comm = SRS-2 Social Communication subscale; SRS_Motiv = SRS-2 Social Motivation subscale; SRS_RRB = SRS-2 Restricted Interests and Repetitive Behaviour subscale; SWAN_Inatt = SWAN Inattentive summary scale; SWAN_Hyp = SWAN Hyperactivity-Impulsivity summary scale.

Table S20.

*Replication Sample – 2 factor, 3 class FMM-3: BCH Procedure Chi-Square Values*

| Test | χ^2^ | *p*-value |
| --- | --- | --- |
| Age – overall test | 1.692 | .429 |
| FSIQ – overall test | 8.500 | .014 |
| Class 1 vs. 2 | 1.697 | .193 |
| Class 1 vs. 3 | 8.462 | .004 |
| Class 2 vs. 3 | 5.316 | .021 |

*Note*. *FMM* = factor mixture model; BCH = procedure for comparing continuous variables across latent classes that takes the probabilistic nature of class assignment into account (Asparouhov & Muthén, 2020); χ^2^ = chi square value; *p*-value = probability value of the χ^2^ statistic; FSIQ = full-scale intelligence.

Table S21.

*Replication Sample – 2 factor, 3 class FMM-3: BCH Procedure Means and Standard Errors*

| Variable | *M* | *SE* |
| --- | --- | --- |
| *Age* |  |  |
| Class 1 | 10.265 | .353 |
| Class 2 | 9.806 | .193 |
| Class 3 | 10.273 | .489 |
| *FSIQ* |  |  |
| Class 1 | 109.284 | 1.603 |
| Class 2 | 106.857 | .864 |
| Class 3 | 101.270 | 2.230 |

*Note*. *FMM* = factor mixture model; BCH = procedure for comparing continuous variables across latent classes that takes the probabilistic nature of class assignment into account (Asparouhov & Muthén, 2020); χ^2^ = chi square value; *p*-value = probability value of the χ^2^ statistic; FSIQ = full-scale intelligence.

Table S22.

*Replication Sample: MANOVA Results – SRS-2 and SWAN Subscales by Factor Mixture Modelling Class Assignment*

| Subscale | *F* value | partial *η*^2^ |
| --- | --- | --- |
| *SRS-2* |  |  |
| SRS_Aware | *F*(2, 415) = 77.244** | .271 |
| SRS_Cog | *F*(2, 415) = 132.783** | .390 |
| SRS_Comm | *F*(2, 415) = 136.662** | .397 |
| SRS_Motiv | *F*(2, 415) = 34.884** | .144 |
| SRS_RRB | *F*(2, 415) = 419.144** | .669 |
| *SWAN* |  |  |
| SWAN_Inatt | *F*(2, 415) = 93.875** | .311 |
| SWAN_Hyp | *F*(2, 415) = 436.650** | .678 |

*Note*. ** = Significant at *p* <.001; SRS-2 = Social Responsiveness Scale (2^nd^ Edition); SWAN = Strengths and Weaknesses of ADHD and Normal Behaviour; SRS_Aware = SRS-2 Social Awareness subscale; SRS_Cog = SRS-2 Social Cognition subscale; SRS_Comm = SRS-2 Social Communication subscale; SRS_RRB = SRS-2 Restricted Interests and Repetitive Behaviour subscale; SWAN_Inatt = SWAN Inattentive summary scale; SWAN_Hyp = SWAN Hyperactivity-Impulsivity summary scale.

Table S23.

*Replication Sample: Brown-Forsythe Test Results – SRS-2 and SWAN Subscales by Factor Mixture Modelling Class Assignment*

| Subscale | *F** value | *p* value |
| --- | --- | --- |
| *SRS-2* |  |  |
| SRS_Aware | *F**(2, 153.777) = 61.516 | < .001** |
| SRS_Cog | *F**(2, 145.673) = 96.230 | < .001** |
| SRS_Comm | *F**(2, 144.811) = 94.696 | < .001** |
| SRS_Motiv | *F**(2, 138.316) = 24.590 | < .001** |
| SRS_RRB | *F**(2, 139.992) = 326.017 | < .001** |
| *SWAN* |  |  |
| SWAN_Inatt | *F**(2, 171.383) = 82.513 | < .001** |
| SWAN_Hyp | *F**(2, 111.719) = 294.728 | < .001** |

*Note*. ** = Significant after Benjamini-Hochberg correction for false discovery rate of .001; SRS-2 = Social Responsiveness Scale (2^nd^ Edition); SWAN = Strengths and Weaknesses of ADHD and Normal Behaviour; SRS_Aware = SRS-2 Social Awareness subscale; SRS_Cog = SRS-2 Social Cognition subscale; SRS_Motiv = SRS-2 Social Motivation subscale; SRS_Comm = SRS-2 Social Communication subscale; SRS_RRB = SRS-2 Restricted Interests and Repetitive Behaviour subscale; SWAN_Inatt = SWAN Inattentive summary scale; SWAN_Hyp = SWAN Hyperactivity-Impulsivity summary scale.

References

Asparouhov, T. & Muthén, B. O. (2020). Auxiliary variables in mixture modeling: Using the BCH method in Mplus to estimate a distal outcome model and an arbitrary secondary model. Mplus Web Notes.

Clark, S. L., Muthén, B., Kaprio, J., D'onofrio, B. M., Viken, R. & Rose, R. J. (2013). Models and Strategies for Factor Mixture Analysis: An Example Concerning the Structure Underlying Psychological Disorders. *Structural Equation Modeling,* 20**,** 1070-5511.

Clark, S. L. & Muthén, B. O. (2009). Relating Latent Class Analysis Results to Variables not Included in the Analysis.

Fornell, C. & Larcker, D. F. (1981). Evaluating Structural Equation Models with Unobservable Variables and Measurement Error. *Journal of Marketing Research,* 18**,** 39-50.

Hair, J. F. (2014). *Multivariate data analysis*: Harlow : Pearson Education Limited.

Kline, R. B. (2016). Principles and practice of structural equation modeling (4th ed.). Guilford Press.

Satorra, A. & Bentler, P. M. (2010). Ensuring Positiveness of the Scaled Difference Chi-square Test Statistic. *Psychometrika,* 75**,** 243-248.
